# Supplementary figures and images for: A new neuropeptide insect parathyroid hormone iPTH in the red flour beetle Tribolium castaneum
Source: PLoS Genet. 2020 May 4;16(5):e1008772. doi: 10.1371/journal.pgen.1008772 (PMC7224569; doi:10.1371/journal.pgen.1008772)

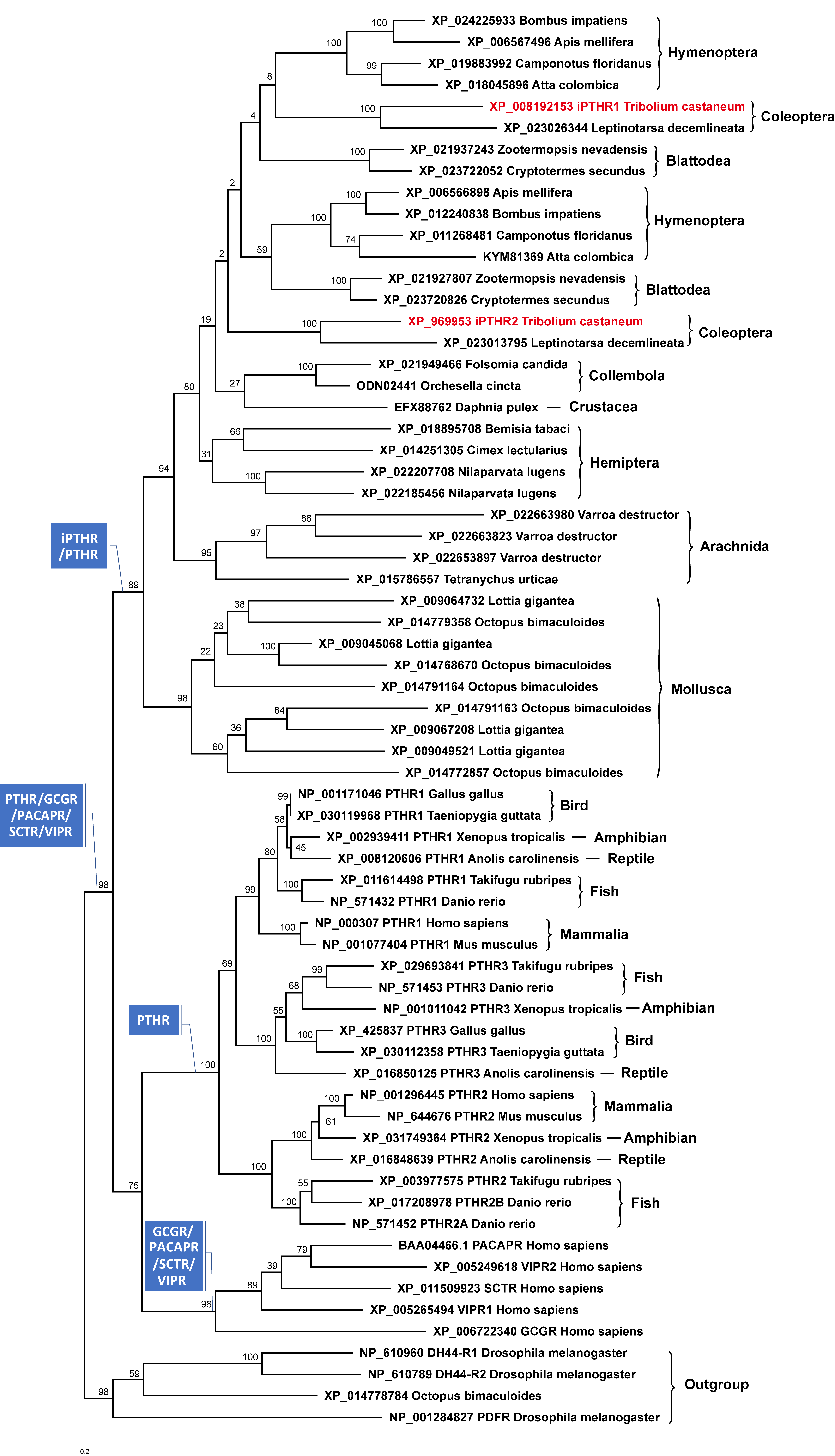

Supplement: S1 Fig — (TIF) [file pgen.1008772.s001.tif]

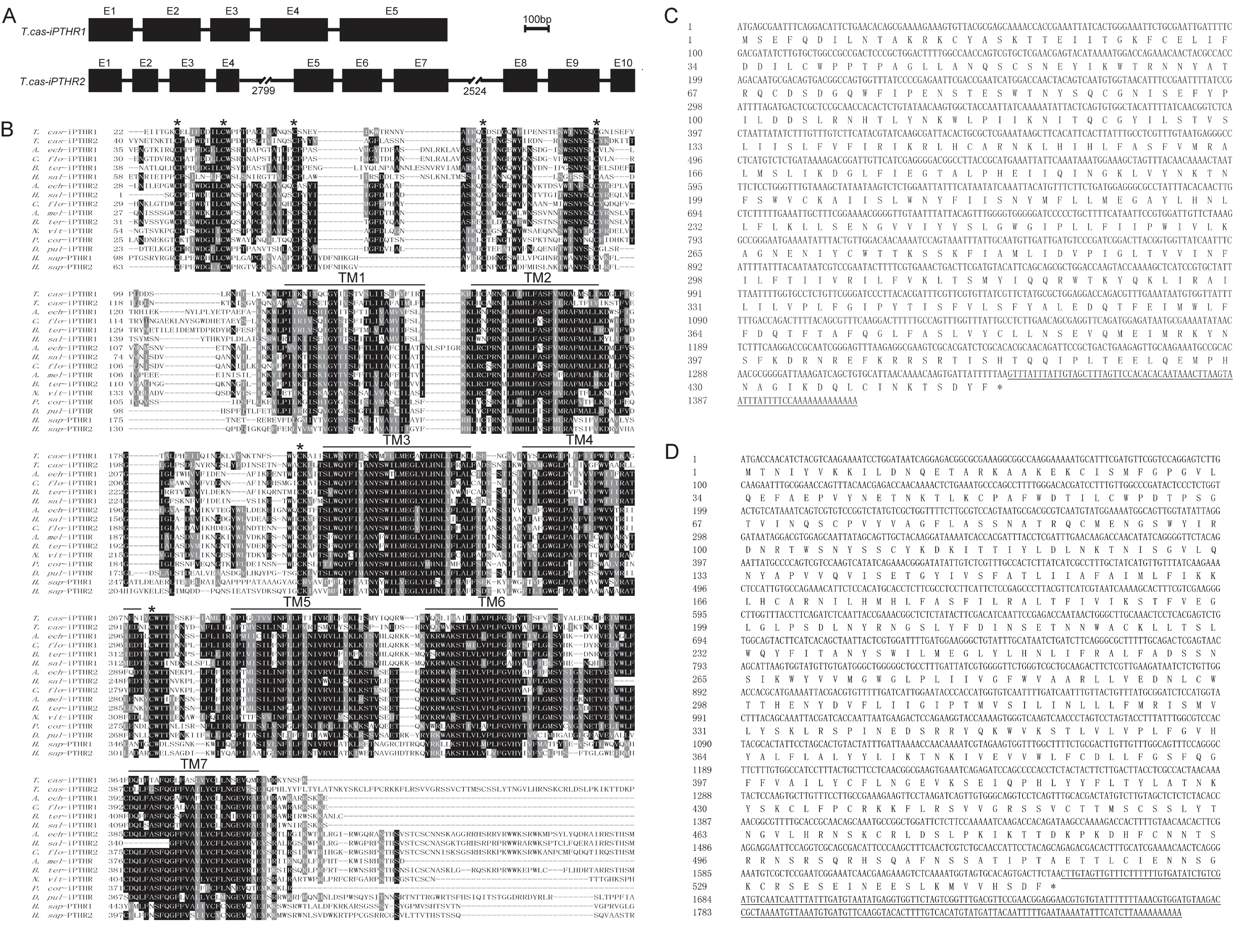

Supplement: S2 Fig — (A) Gene structure of the Tc-iPTHRs. The blank boxes represent the exons, and the line represents the introns. (B) Amino acid sequence alignment showing the similarities among PTHRs of invertebrates and human. Inverted letters with black and gray background are identical and similar amino acids in the sequence alignment with the 50% majority rule. Seven transmembrane regions have been marked above the sequence alignment. Star “*” indicates the conserved cysteine residues. (C-D) The sequence of the Tc-iPTHR1 and Tc-iPTHR2, respectively. The underline is the 3' UTR sequences. (TIF) [file pgen.1008772.s002.tif]

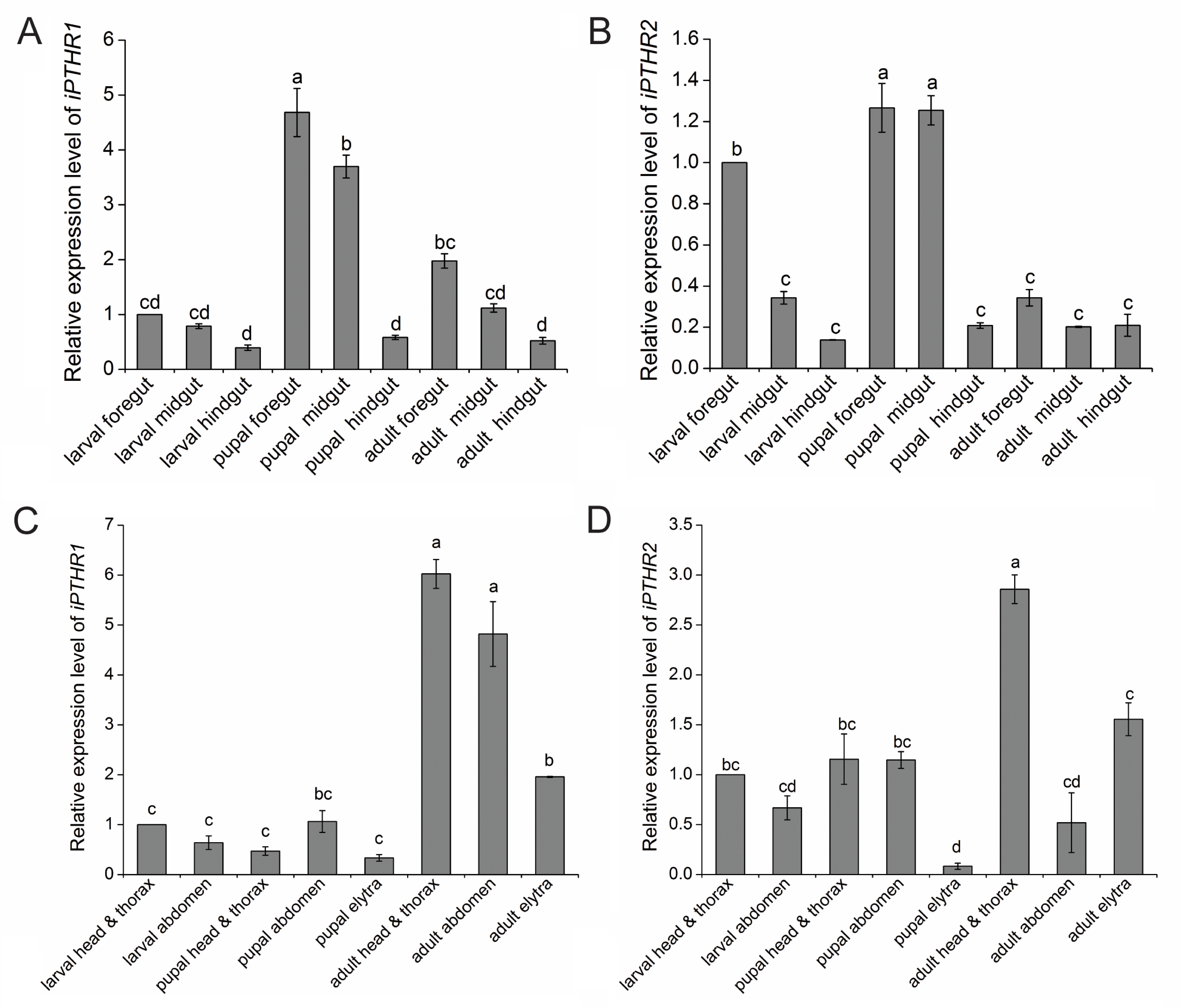

Supplement: S3 Fig — These data supplement Fig 3 in the main text. (TIF) [file pgen.1008772.s003.tif]

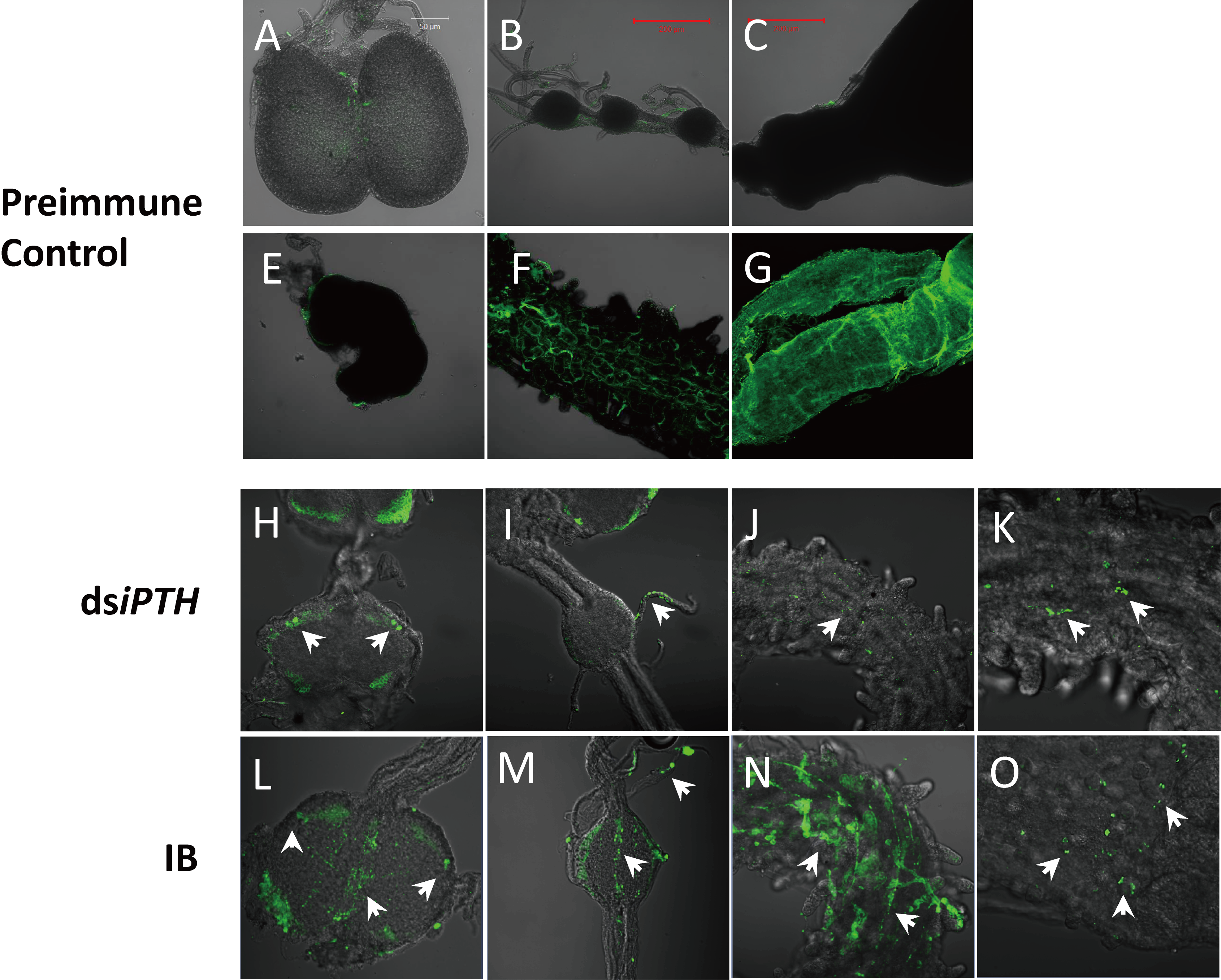

Supplement: S4 Fig — Preimmune serum was used as the negative controls of Tc-iPTH for immunohistochemistry and did not show any specific staining patterns. (H-O) Immunohistochemistry of Tc-iPTH in the late pupal stage with (H-K) or without (L-O) larval injection of dsRNA. Please note that compared to the IB pupae, the immunoreactivity in the dsiPTH pupae was significantly reduced, but still displayed remaining immunoreactivities for the Tc-iPTH. (TIF) [file pgen.1008772.s004.tif]

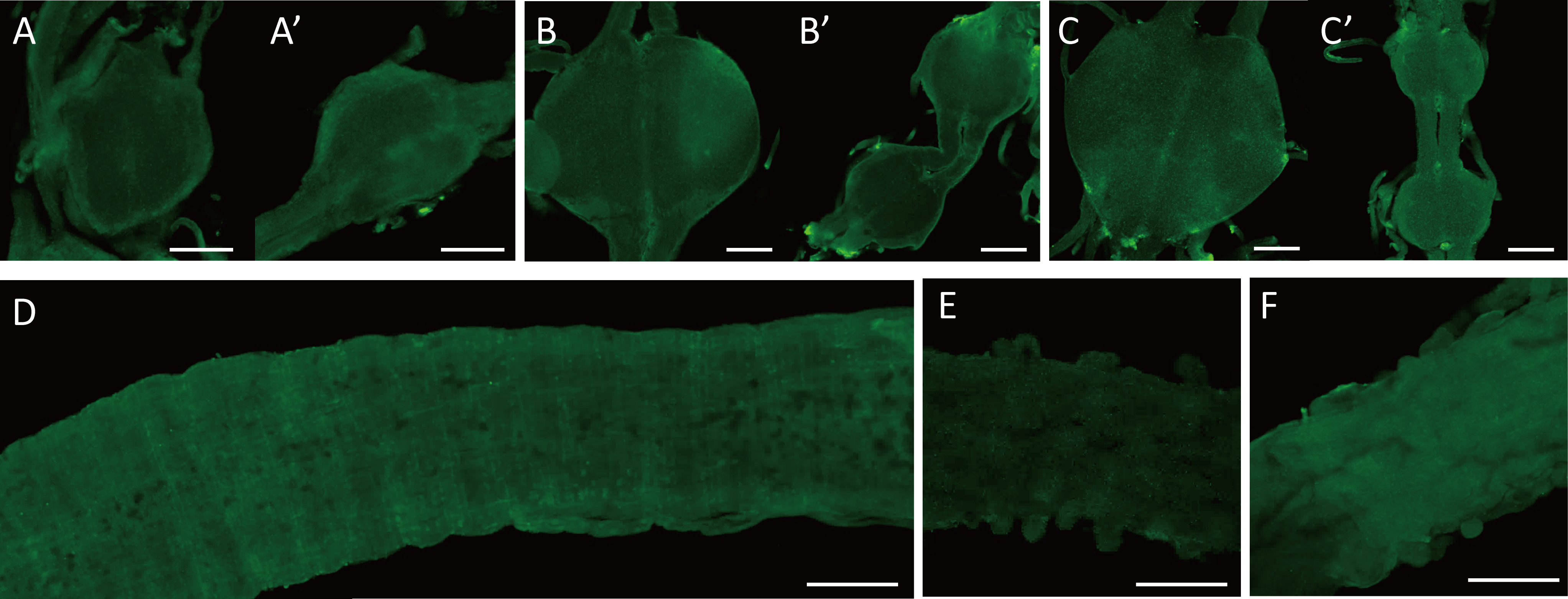

Supplement: S5 Fig — Negative controls of the immunohistochemistry of anti-Tc-iPTHR1 antibody staining in the central nervous systems and gut in larval (A, A’, D), pupal (B, B’, E) and adult (C, C’, F) stage. Preimmune serum was used as the negative controls of Tc-iPTHR1 for immunohistochemistry and did not show any specific staining patterns. (TIF) [file pgen.1008772.s005.tif]

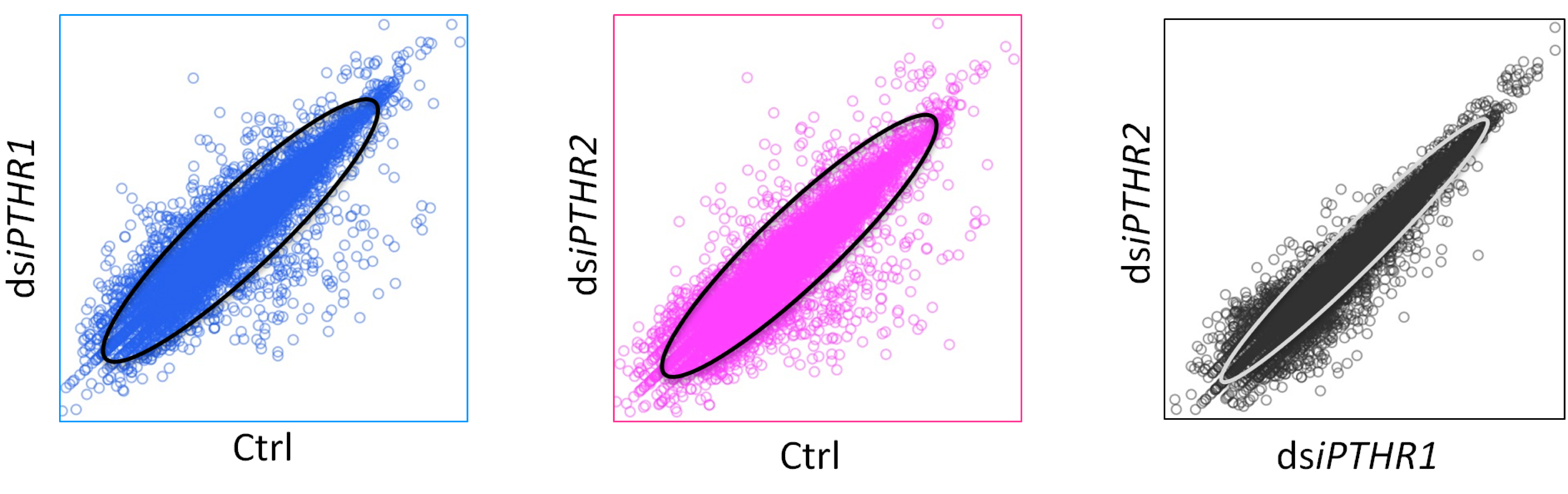

Supplement: S6 Fig — (TIF) [file pgen.1008772.s006.tif]

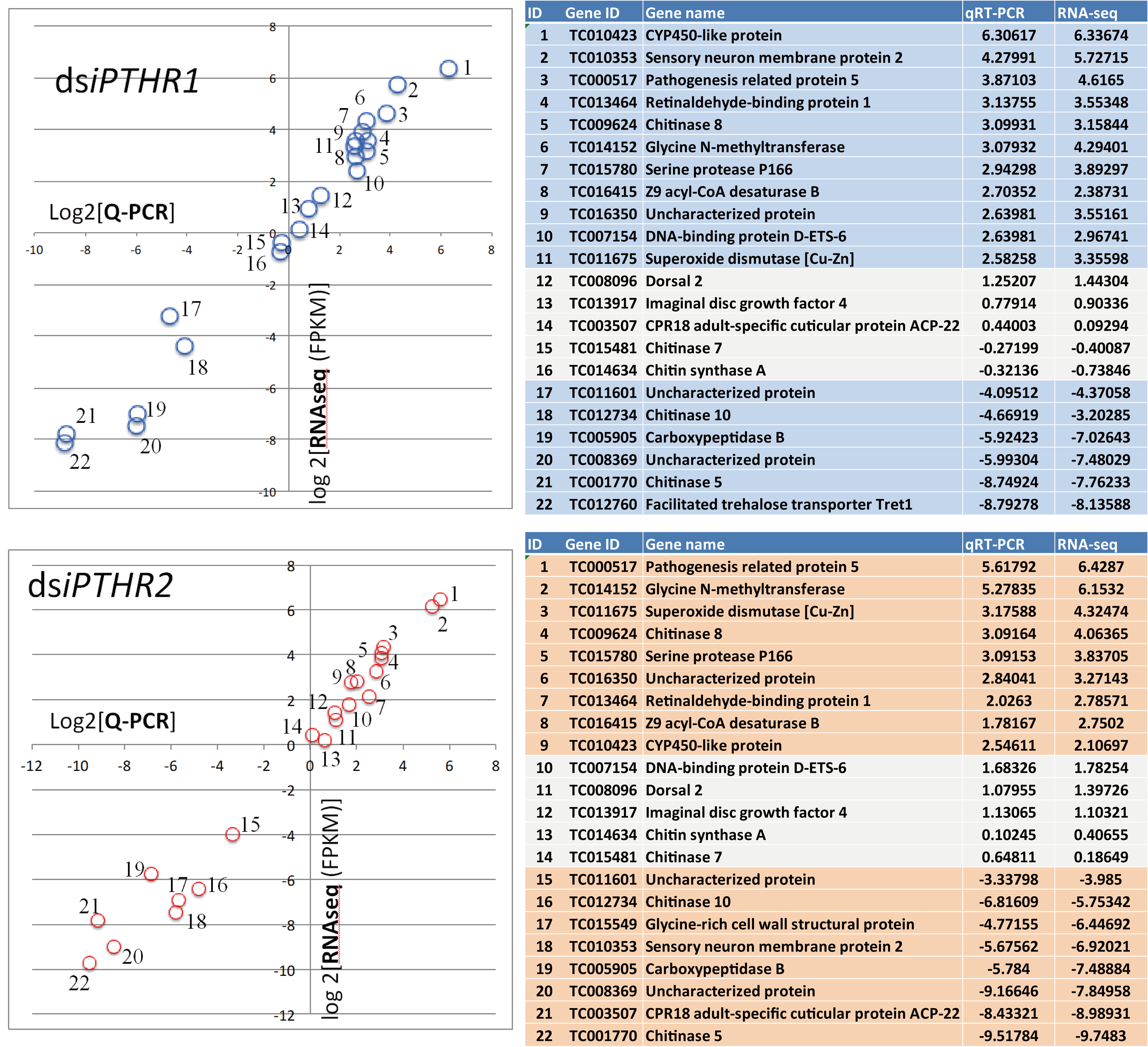

Supplement: S7 Fig — Twenty-two randomly selected differentially expressed genes between the control and dsiPTHR1 or dsiPTHR2 as determined by RNA-sequencing and Q-PCR. (TIF) [file pgen.1008772.s007.tif]

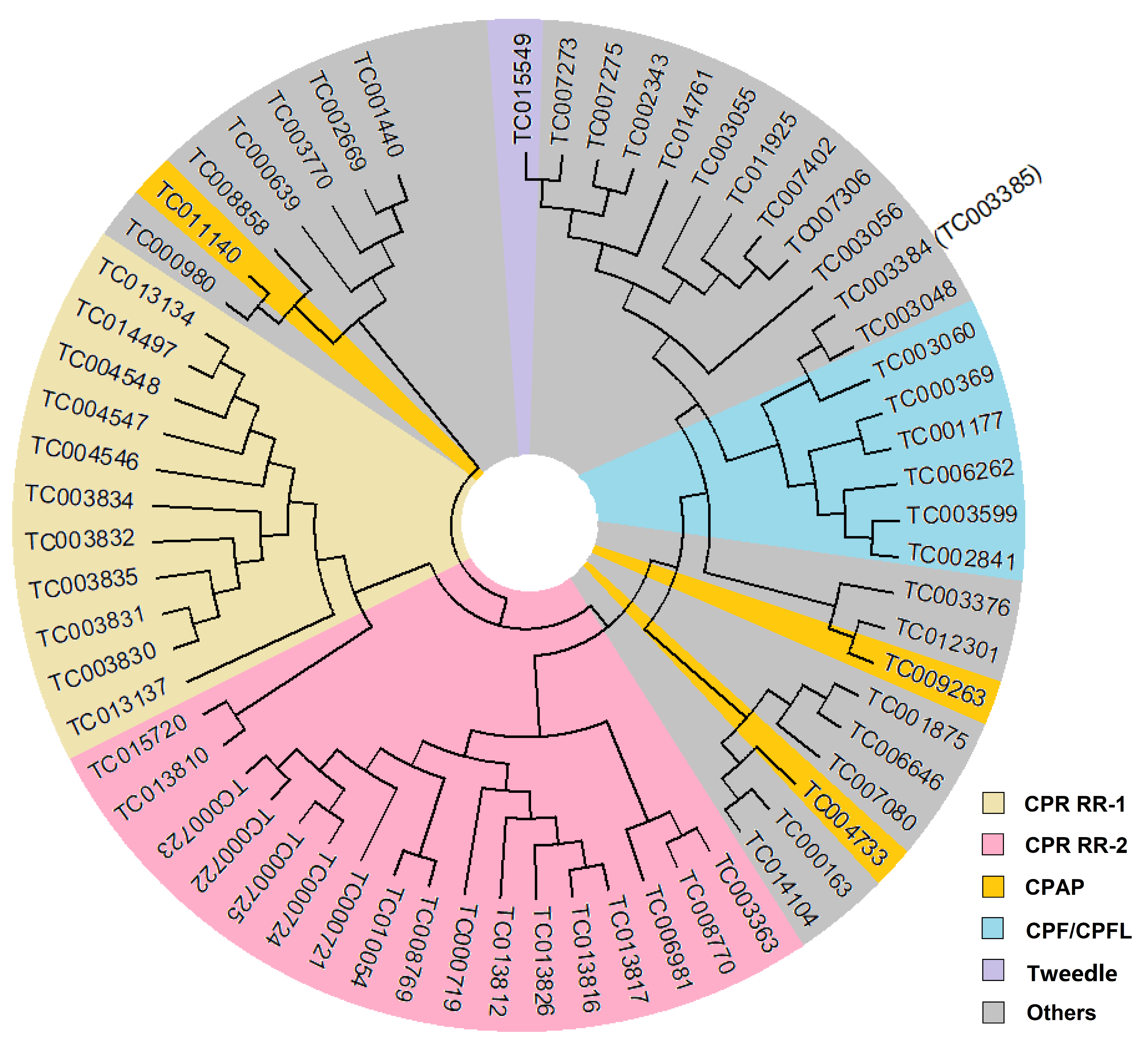

Supplement: S8 Fig — (TIF) [file pgen.1008772.s008.tif]

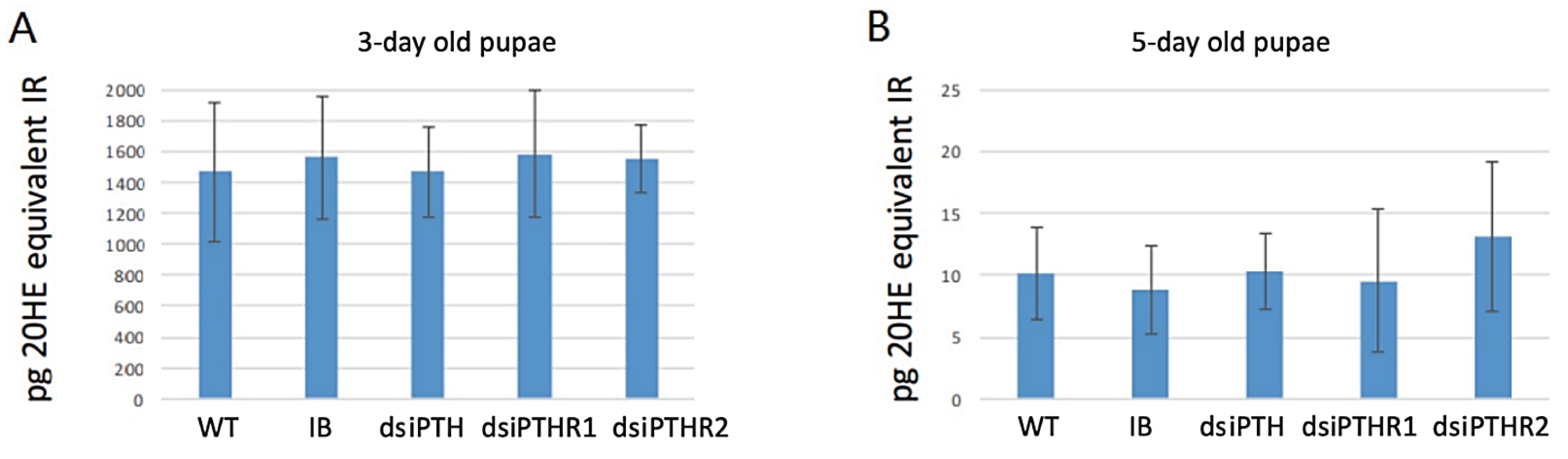

Supplement: S9 Fig — (A) Immunoreactivity for 3-day-old pupal stages for no injection, buffer injection, RNAi for Tc-iPTH, for Tc-iPTHR1, and for Tc-iPTHR2. (B) Immunoreactivity for 5-day-old pupal stages. Error bars are for standard errors of 3 biological replications. (TIF) [file pgen.1008772.s009.tif]
