# Supplementary material for: A new neuropeptide insect parathyroid hormone iPTH in the red flour beetle Tribolium castaneum
Source: PLoS Genet. 2020 May 4;16(5):e1008772. doi: 10.1371/journal.pgen.1008772 (PMC7224569; doi:10.1371/journal.pgen.1008772)
Supplement: S1 Table — (PDF) [file pgen.1008772.s010.pdf]

Table S1. Fasta files showing PTH sequences captured in GeneBank database searches.

NEMERTEA
>Notospermus geniculatus
MEKGHVILFVCVFATILITVSSKSIEHDIHREKRASSDQRVAELQALIALSRGRGLVGHGHIDPYMAGKRKR
ADLLSRLNPSEKDRLLETLIQRVLAAKEAEES
BRACHIOPODA
>Lingula anatina transcript 1
MQKTHSCAALLLATLAFMMLAFTSAKSISSVQHSRHRVRRQGGDMTIADRLAWLSQNIRQPVGCSDAACG
FVDTDKIGKRKRESTDYYYNNGESSFSDLRKERILQRLTQLLIEGNQ
>Lingula anatina transcript 2
MQKTHSCAALLLATLAFMMLAFTSAKSISSVQHSSRVKRQWADARMAELLPLMGLMRGGPGSVAHGMV
DPAVNGKRKRESTDYYYNNGESSFSDLRKERILQRLTQLLIEGNQ
PHORONIDA
>Phoronis australis transcript 1
MRSRDFVVFAVLFVIVVVFSSAKSLPLSLRRLQRRQADVRLADMLASINQQGASPTGCAEVGCGLNLDEV
GKRSVEKQRSPMIYPDERDRETEIVYRLLRRLAHEMDERL
>Phoronis australis transcript 2
MRSRDFVVFAVLFVIVVVFSSAKSLPLRSKRSNADQRLAEMQAFLSLYGHGSTMPDPLRAGKRSVEKQRSP
MIYPDERDRETEIVYRLLRRLAHEMDERL
MOLLUSCA
>Sepia officinalis gene 1 transcript 1
MTRNLLVVVLVAILVSTLANGRYIADTKLSSYKRTSSDQRIAELQALIALSNTIGHGQVNPEEIGKKKRTDT
NSVDFRRSLLVERLLRLAAEGLVNSV
>Sepia officinalis gene 1 transcript 2
MTRNLLVVVLVAILVSTLANGRYIADTKLRSKRQTGDLKAAAYQAWLALGRTLPPDCPEVACGVVDVEA
SGKKKRTDTNSVDFRRSLLVERLLRLAA EGLVNSV
>Sepia officinalis gene 2
MAPLQYILPLLLVLPIIAAWNPVLRSNEINSIRRAALLKHNEDSSDFGVYQRNGRDASDLQRAFSDYLKSSV
EDSKSAWSDPCRLNLGGRCATEIASDLVKAWHYLNSSNSPGRKRRDVREALRTILRHSAAAAAAAAAADN
R
>Lottia gigantea gene 1 transcript
MNNLQVQILTVIFSLCMALFTDGASLSHLRNKRQTADVRTTEYLARLGLGRGLNSYGCRDIACGVVDIYRS
GKKKRGDTGKLGDSSVEVGDVDLLRALIRQSLQENSV
>Lottia gigantea gene 2 transcript 1
MPRFGVALCSIVLFVVLVSTLTESKSLSRQKRQLADLKTSELSALISLGGRYAPRGCKDVACGLVDIFKSGK
RNGNNEYLRDLSLEERYQLIRNILQRAAERKASFEYVN
>Lottia gigantea gene 2 transcript 2
MPRFGVALCSIVLFVVLVSTLTESKSLSRRKRQADSGLMQLLTDRKTNDCYFGLGCGVEDVFASGKRNGN
NEYLRDLSLEERYQLIRNILQRAAERKASFEYVN
>Lottia gigantea gene 2 transcript 3
MPRFGVALCSIVLFVVLVSTLTESKSLSRVRRSGADQRIAELQALIALTSHGGMVAHGQFDPLRIGKRNGNN
EYLRDLSLEERYQLIRNILQRAAERKASFEYVN
>Crassostrea gigas gene 1 transcript 1
MLRTQILASVCCLLIAVSSLVDSRFLDQDEEARFKRQTADMRLSELHAIREILNRLPTGCSQYACGLVDIFRS
GRRKRAAEWLSKTFSNGKNSADEETSH
>Crassostrea gigas gene 1 transcript 2 transcript found
MLRTQILASVCCLLIAVSSLVDSRFLDQDEEARYDKRSWSDQRMAEIKALLGLKNQGNVIAHGLYDPYKIG
RRKRAAEWLSKTFSNGKNSADEETSH
>Crassostrea gigas gene 1 transcript 3 transcript found
MLRTQILASVCCLLIAVSSLVDSRFLDQDEEARFMRKRTSSDQRIAELQALIALVHGRGNVAHGQLDPALIG
RRKRAAEWLSKTFSNGKNSADEETSH
>Crassostrea gigas gene 1 transcript 4 transcript found
MLRTQILASVCCLLIAVSSLVDSRFLDQDEEARYDKRSWSDQRMAEIKALLGLKNQGNVIAHGLYDPYKIF
MRKRTSSDQRIAELQALIALVHGRGNVAHGQLDPALIGRRKRAAEWLSKTFSNGKNSADEETSH-
>Crassostrea gigas gene 2 transcript found, except first exon different

MTYRLFPCLGDRSSFLDGLIGVLVTVKWVFTLSDATCDVGRMRERMGVRRQNADVKATEYFARLALERM
PTDCLQIGCGLVDLQESGKKKRTDTQSSPYHKQRLLKIVQLLIPKEQ
>Octopus bimaculoides transcript 1
MTRHILILSIVGMVLVSLVNARYIPEEELRSKRQLADMRAAEYRALMALSRKITCTQVACGLVDIEKSGKK
KRSDISSDDIRRSILAQKLIQFAAERLAKTE
>Octopus bimaculoides transcript 2
MTRHILILSIVGMVLVSLVNARYIPEEELRSKRQTGDIKTAEYQAWLALGGIVPPGCVEVACGVVDVHASG
KKKRSDISSDDIRRSILAQKLIQFAAERLAKTE
>Octopus bimaculoides transcript 3
MTRHILILSIVGMVLVSLVNARYIPEEELSVKRTSSDQRIAELKALLAMSRVVGHGQIDVDAIGKKKRSDISS
DDIRRSILAQKLIQFAAERLAKTE
ANNELIDA
>Platynereis dumerilii
MPNSQVLIATVIFCVAVSCVCSKSIQVFNRHNERLKRQIGDLQIAHHQAQIGLKHWPKGCVEIGCGLFDIAA
SGKRKRSYSNNVEDEFLNNENELRRESNRLVLLQRIADELMRES
>Pomatoceros lamarckii transcript 1
MQTSQVLFVSVLLVVFACVFARSVPDYRNRVKRQFADVLMSKHQANIGLRNLGPKACKEFACGLVDIAAS
GKRKRSDEYAMSRDEFDDYSRLALLERVLDTIMEDEDS
>Pomatoceros lamarckii transcript 2
MQTSQVLFVSVLLVVFACVFARSVPDYREKRAGSDQRLSELQALISLKEGKVGHNLRVGDIWKRAGSDQR
LAELQALIALSNDGYDVIGHGLLDPTRAGKRKRSDEYAMSRDEFDDYSRLALLERVLDTIMEDEDS-
>Capitella teleta transcript 1
MPGILKLLVVCIFSILLVVGPISARGVPETDVGTRRTRRQMGEVHLGAHRARLSMKQQLTPNGCREVGCGL
IDFALSGKKKRSEVLDLGDQSGYMESAMRRSRLIRKLLTQLAERDLL
>Capitella teleta transcript 2
MPGILKLLVVCIFSILLVVGPISARGVPETDVGTSRTKRANSDQRMAELQALIALSNGGGETLGHGAIDPYS
AGKKKRSEVLDLGDQSGYMESAMRRSRLIRKLLTQLAERDLL
TARDIGRADA
>Hypsibius dujardini
MPAFSRVVLWTIVVLYLSQLVMSRSIPLHLSLREKRSGISDQRLAEIEALIAMAKAGRHHYGGGKLHGNSPF
GGYNGVNPADVG
ONYCHOPHORA
>Euperipatoides rowelli
Present, partial exon coding PTH from genome
XIPHOSURA
>Limulus polyphemus gene 1
MSTESRKHTKVMITLIVLLYFSEALPAARERRSGISDSRLAEIETRLALSKPVAYGIFDPVKIGKRTFRRFQPP
PGDFNNVDLVYAWLNMKNADYLKEPINVVKTPY
>Limulus polyphemus gene 2
MMNQTSTLFMVIFMAMLFLAAGRPYLRERRSGISDQRLAELETLVNISKLRSSVYGLVPAVAFGAFDPARL
GKKRRSNKIPKLMHRLKAPTSQSYTSKNYIKKE
>Limulus polyphemus gene 3
MNNISMKTMIFLVALLLLKVSLAHPQARIKRSGISDTRLAELETQVSLNRPRSGSVAYGIFDPSKIGKRTFHQ
FRQFPEPTDIVYDDPDYIWNTPKDNYQQEAIIFKNPFLADGAEWLSEILSLAKIKTRPNSKKEDIA
>Limulus polyphemus gene 4 missing C-terminal
MALLARSVPCLFLFLLMFSLAVGRPHDRERRSGISDQRLAEIETLIAMYKLNRNRDKEVTGSVAFGAFDPTI
LGKKKRSYSVSAVENKYGGPLRADNTKNEEDSETSSTAELSSLTSKDVPDHSKMDILHLRKILAKLRTINEQ
IQHDNTY
>Limulus polyphemus gene 5 missing C-terminal
MALWRKINPTLFVLTFLIMFCLAAGRPYHREKRSGISDQRLAEIETLIAMSKYARNRAKEVTSPIAFGVFDPT
NLGKRKRSYGMSSAEGKYDEQFPQLYTFPTYNTEKYGNSEPPSLLNTDLSHLSSLDILHLWRLFFKLQIRNE
NQLEKND
>Limulus polyphemus gene 6
MFNSQPTSVKLLQKMGETSDKKKFAKAKYDEGMTMPKSTETRQRKSGISDARLAELETQLSLNKMRSGPV
AHGIFDPAKIGKRRFRQVAPFRELTEINADDTVYVWIIMKLESNLSSEELDIFENTAKA
>Limulus polyphemus gene 7

MCTRMFLFYFLRILYIFKNVHIKIIHRSTERRSGISDQRLAEIETLIAMYKLNRNRDKEVTGSVAFGAFDPTIL
GKKKRSYSVSAVENKYGGPLRADNTKNEEDSETSSTAELSSLTSKDVPDHSKMDILHLRKILAKLRTINE

ARACHNIDA

>Ixodes scapularis
MVRSHCRRHREAMSTKLAFLVIFCAVVLLPAIVQCRSLYNRSRRSGISDQRLAELETLAGLKSLRHRLKGIS
FPVAYGLVDPNKIGKRKRSFESLEDSQQPQQQQGGEYQTGEDGGLEDLLSGMPTPDMVSRMRPSSDQLLRI

>Stegodyphus mimosarum gene 1
MCRISLAVTIFCFLVTLVSCHIRERRSGISDQRLAEIETLLAMENSQRGHNNVAYGVIDPLSLGKRKRSHDKG
IIPMRLEDFPNDNLRESRLKLPYFRGESLMYPRRLPERIYNRFQVNDLENRDMQADDASSK

>Stegodyphus mimosarum gene 2
MCRISLAVPIFCFFVVFVSCDFRERRSGISDQRLAEIETTLAVQNSQRRNNNVAYGLIDPMALGKRKRSYDK
DIILVQLENLPVEELRESRLKLPYFRHKALTYPRRLPSREYNIFISNDLENRDLQMDDDLSK

>Stegodyphus mimosarum gene 3
MCRISLAVTIFCFLVTLVSCHFRERRSGISDQRLAEIETLLAMEKSQSGSNNVAYGLIDPLSLGKRKRSYDKD
IFPIQLEEDFPADYLRESRLKLPYFRHEALTYPRRLLDRVYNRFIANDLENRDLQADDDSSK

>Latrodectus hesperus
MSKISLAIAAFCFLFTFVSCHFRERRSGISDQRLAEIETLMALAKQGSNKQPDYGYGIIDPLILGKRKRSYDM
ESSNQLEDFPLDIFHEARAKYPSNRYESLMHPYRFPERLFRFRTNDLENRDLQVNDDTAK

SCORPIONES

>Centruroides sculpturatus gene 1
MRLHRGSSLLTFIFFLFILTITFSMPHYSFRDKRSGISDQRLAELETLINLAKQKNRGRPPIAFGVIDPLKVGKR
KRSNDVTEMDDLRELFDDPLKEKEYAENARFWDLMTDLRRYTN

>Centruroides sculpturatus gene 2
MNREKNIFLSVRLLGTFLLLSQVFAHRLNRERRSGISDQRLAELETLLALSKFKSFHTPVAYGLLDLEKIGKR
KRSIEDAKEEEKKRFYPVDFRGEDTTPTKPRRKNSAPSERNEMRYDGRRR

>Mesobuthus martensii gene 1
MRLHRGSSLLTFIFLLFILTITFSMPHYSFRDKRSGISDQRLAELETLINIAKQKNRVRPPIAFGVVDPMKVGK
RKRSNNNNLDLEEYTELFNDPLKEKEYAENLRYWNLITGLRRYSD

>Mesobuthus martensii gene 2
MNQEKNILIFIRVLGILLLLTHGFAYRLNRERRSGISDQRLAELETLLALSKLKTFHSPVAYGLLDLEKIGKR
KRSIEYLDEEEKPFYPVDFGGGETKYGKSDSETWIPLWRKKVDFNRSE

MYRIAPODA

>Strigamia maritima
MAPTKSHVALLILIISAMTVQSASTEREKRSGISDQRLAELETLLALSKMKGKLVTVPVAFGVIDPDKIGKR
NHQYEMPALDDDVERNDEVWLRQFQQRFDQYKKA

COLLEMBOLA

>Orchesella cincta gene 1
MMGMSMAAYWMLIIVTIASVTGKPASTMRTHKRQSDQRVAELETLLALQKYGAHHFLRAQSSDGGGFFP
GYGIIDFNKIGRKKKSSSQSHEALDWALPSWLAPNPSSDTRLSSRNWKETLDPVTTGSDPVEGDDDEN

>Orchesella cincta gene 1
MNNKVFASFFVLVIIMLIASLTSARPSNQLSLSRQKRQSDQRIAELETLLALQKYGRHRFRMDRKKGVGAD
GEHPPGYGIIDFHMLGRKKKSTRFSPGELESSLKGVPQPDLLSSYHPEHQLDSMESSAEESVFPVQREHDDK
SRQEQDAALPFLYVEKK

EPHEMEROPTERA

>Ephemera danica
MRRNSAFLMCSVVVVCLALLVPDLVQARPHRQKRVSDQRLAELETLLALAKMKGKLITVPIGFGRVDPTK
IGKRRRRSQQELDENRLELPFDSMDDSAEERSGSSPQYEDLLRQYLLAWARDQESI

ODONATA

>Ladona fulva
MGDKHHGATSSMGRIILIVLLLFLASEGGARPADRRKRVSDQRLAELETLLALSKTRGKLVTVPVGFGRIDP
QRLGRRKRTLIGENDRDEEECFCCTWFRQHQDNLQQPPAGCDHSHYLFHRTRGLKNGSSNLVHFALDKEA
LQTPNEVDYINVL

PHASMIDA

>Clitarchus hookeri
MNMSFATRAVAVLLIVVLVADQRAEGLPYRRKRTSDQRLAELEAYIGMSKMKGQMITVPIGFGRVNLDEI
GRRRRSVRTYPLQDVMDSPRPRDSSEESLDDLLHDIERSRPYQWDAY

BLATTODEA

>Periplaneta americana
SRIIVIVFVVVLATMLALPQTHGRPYRQKRVSDQRLAELETYMALRRMAGKLVNVPVGFGQVDPAKIGRR
RRRSAELLLQELLNSHDDSEATADADAMLADNNESDEDVRDLVGPHRPSQQWLPEWSRRVQV-
>Blattella germanica
MVIKTLAFAHFVIIITVEALLTGLHGTEPQKRVSDQRLAELETYIALRNLAGKIVTVPVGFGQVDPAKIGRRR
RRSAELLLQELLNSPTAHEDAIAEAADAILSDNNVESEEELREAHRPTQQQWLPEWSRRVQVSFKTQIQQ
ISOPTERA
>Zootermopsis nevadensis transcript 1
MRRGTIVVLWVVLLVALLTVSQAHARPHRQKRVSDQRLAELETYMALRKMAGKLVTVPLGFGQVDPAKI
GRRRRRSLEVLLQELRNPLTQDDRDNTADANANVNAMLADNSESDEDVRDYVESHQSLPEWYHAAQV-
>Zootermopsis nevadensis transcript 2
MRRGTIVVLWVVLLVALLTVSQAHARPHRDKRQTDFVVKQYITQLNRRQAVTIIVPCAGLNTTSCGRRRR
RSLEVLLQELRNPLTQDDRDNTADANANVNAMLADNSESDEDVRDYVESHQSLPEWYHAAQV
ORTHOPTERA
>Locusta migratoria
MSRIACSLLLAAVVAALAASECNAMPADNHRVKRMSDHRVAELQTLLSMGKMGGKVVTHKVGYGQVD
PMKVGRRRRSDPRMLSRLQQLLLSAAAGDSHEQFPEQEDEQQLPLEPTEEDERQVLMQLLHGQQEEQSPH
EQLPARLSWWQRPLALQM
PHTHIRAPTERA
>Pediculus humanus
MFYIKFFFILVTAIICSRIIIAKPHHQDFTRLKRTSDQRLAELETLFALSKMNGKLMTVPIGFGKIDPNKLGRR
RRSIVLLNDFTTNNFPKENKLNNNIKR
THYSANOPTERA
>Frankliniella occidentalis
MKTSSIALLAAAAVLVLLAPAVLARPPHRQKRVSDQRLGELEALLALKKLRGKLVTVPVAFGQINPNDYG
RKRRSAPHASAASGHASLLERLLEQAQQDDLLSGLDMYDELPAALEAGRLEGRLEQGPVAEPLHWAAYE
RERP
HEMIPTERA
>Rhodnius prolixus
MYLRLQLTIVLLLVTCYAVHGFRYRQKRVSDQRLAELETLVALSHLKAHLKPFRYGYGSVDPLKVGRKRR
SATNSLLLDKLLEQIEEKRENYLEDELEDDRDEDSDEPTIVQWPPYYWHFRKHHHR
>Halyomorpha halys
MLSRLIILLLAAAIAHGYRFRQKRVSDQRLAELETLVALSNMKGNIKTIPYGYGSVDPIKAGRKRRSSSGALI
DRLLQSIAHEDDDSIGQERPVIQWPPYFWAANDQPE
>Oncopeltus fasciatus (first exon missing from genome assembly)
MLVRILFLVLAVAATVSHGYRFRQKRVSDQRLAELETLVALSNMKGNIKTIPYGFGSVDPNKAGRKRRSPG
VLLDRLMESIAREEEEESNSQERPIIQWPPYYWLEQQTE
>Cimex lectularius gene 1
MYLRLSYVFIAVLSVVVFSDCYFIRYKKGFAISSDHRMNELQAESVLVTQPSSVGFGVLNVTKFGRRRRELT
VVPTQSLERNDFLKEDKESLFLLRLRSPQKL
>Cimex lectularius gene 2
MYLRLSYVFIAVLSVVVFSDCYFIRYKASFPFSSDHRMTEIQTQTRLGNKASSVGHGLYNVSKFGRRRRGLT
VVPTQSLERNDVLNEDKESLFLLLFRSPQKL
>Nilaparvata lugens
MYFRQLFLINVLLFTIFLVNSSCLARPNREKRYSDQRLAELETLYKLQQLRGKFQRGPLDYSKIDPNILGRRR
RSSEVLLDQLRAELDEDRDRNIAPPLDEDFWNSLLI
>Bemisia tabaci
MYSLRIVVFVVVTVIFLCVTNETHGSPFRMKRVSDHRLAELETLLALSKMKGQVMTVPLGYGKVDPERIR
DVSERKRSYTSVETNSHK
COLEOPTERA
>Agrilus planipennis
MDKRVFKVFVFIVVLAMLQIVDARPSRIKRVSSDARLAELETIIGLRNLAGKLVTVPLGMGKVDPQYIGRK
RRSQPRFLDVLFNQSVEDDDGDPVEKEDDYYNR
>Nicrophorus vespilloides
MKKIIFYLGITIVLIVIFVQNVSAKPYSRVKRVSDQRLAELETLVALSKMSGKLVTVPLGYGKFDPKIIGRKR
RSQTSLLLDKLFSTSADENFVIPMPLQDRSNYLQDDYER
>Tribolium castaneum

MKTITFCFFVVLVMSVQNVFAGPRYRLKRVSDAHLADLQSRIALNNKLKGVSVTMPVGGGRIDPGRIGRR
RRSQTRFLDVLFNQSEEDKGDPVELTDYENLIQRLLNLE

HYMENOPTERA

>Apis mellifera
MAGLFTTLLVLCALNVILIPDTIYARSFPLLSRQKRISDQMLAEADTRSGLSNVKGMVKTVPVGFGVFNLEQ
IGRRRRSTINNKLETLKRIMQIINDNPELLDEDNVLSPQVKLKENQSDEYSLI

>Bombus terrestris
MAANTSLLVTLMVLCVLNVILMPGTTHARPPSLLSRQRRISDQRLAEIETLLGLENVRGKVVTVPVAFGVL
DPDKIGRRRRSATNNRLETLQRMMKIIGNRPDTFNENMLGERMLSLPAKLKESRLGVNDEYSYI

>Polistes canadensis
MTKYEMCITSRPLVTLLVLSVLVTILIPEPTDARPTDTVISRRKRVSDQRIAELETLLGLSTKIKGKVIFVPVA
YGVIDPAKVGRKRRSSSDDSFNKLREILQIDRQDSIFLPEENNEILSLPLKDANNHLEIERQY

>Nasonia vitripennis
MSRYAASALAVAGIVLCVVLLVQVTEARPSALLSRPKRVSDQRLAELETLIALQKMRGKLVTVPIGFGKVD
PMKIGRKRRSSTETSWQELNRILRSIVEEDAELSPNDRIRFARLFSSRWDEVRNQDDTPTANDRGEDGALY

>Athalia rosae
MSSSAGSRMTLLSTFGAVILVLVLLAAENDARPSGRISRQKRVSDQRLAELETLLALERIRGQLVTVPVAFG
RVDPAKIGRRRRSSVEGGFERLQRILQAAAQDSQSVGQESDESASSIWDDIQDRREEETEDQFI
