# Supplementary material for: A new neuropeptide insect parathyroid hormone iPTH in the red flour beetle Tribolium castaneum
Source: PLoS Genet. 2020 May 4;16(5):e1008772. doi: 10.1371/journal.pgen.1008772 (PMC7224569; doi:10.1371/journal.pgen.1008772)
Supplement: S2 Table — (PDF) [file pgen.1008772.s011.pdf]

Table S2. Primer sequences used in this study Tc-*iPTHRs*, Tc-*iPTH*, the internal control gene Tc-*rps3*, and other genes tested for the Q-PCR.

Primers of Tc-*iPTHRs*, Tc-*iPTH* and the internal control gene Tc-*rps3*

Name	Remarks	Sequence(5'-3')	Primer length	Product length	Position
Tc-*iPTHR1* (TC008110)	Full length clone	ATGAGCGAATTTCAGGACATTC	22	1341bp	1-22
		AAAATAATCACTTGTTTTGTTA	22		1320-1341
	Q-rtPCR	GTTTGTCTTCATACGTATCAAGCGAT	26	220bp	411-436
		CCAGAGACTTATTATAGCTTTACAAACCC	29		602-630
	RNAi	TAATACGACTCACTATAGGGGGAAATTCTGCGAATTGAT	40	457bp	76-95
		TAATACGACTCACTATAGGGCCTCATTACAAACGAGGCAA	40		473-492
Tc-*iPTHR2* (TC010267)	Full length clone	ATGACCAACATCTACGTCAAG	21	1647bp	1-21
		GAAGTCACTGTGCACTACCAT	21		1627-1647
	Q-rtPCR	GCGGATCTCCATGGTACTTT	20	233bp	975-994
		AAAGGATGGCCACAAAGAAG	20		1188-1207
	RNAi	TAATACGACTCACTATAGGGTTTCTTGCGTCCAGTAATGC	40	476bp	238-257
		TAATACGACTCACTATAGGGAAGCCCAGTTATTGGTCTCG	40		654-673
Tc-*iPTH* (TC015123)	Full length clone	TCGCAGTGAGCGTGTTATTT	20	404bp	69-88
		TTCATTCCGATTCAACCATT	20		453-472
	Q-rtPCR	TTTGGCCGATCTTCAGTCCC	20	167bp	198-217
		CCTTATCTTCCTCCGATTGGTT	22		343-364
	RNAi	TAATACGACTCACTATAGGGACCATCACATTTTGTTTTTC	41	296bp	103-123
		TAATACGACTCACTATAGGGCTTCCTCCGATTGGTTG	37		342-358
Tc-*rps3* (TC008261)	Q-rtPCR	TCAAATTGATCGGAGGTTTG	20	260bp	320-339
		GTCCCACGGCAACATAATCT	20		560-579

The underline sequences is the T7 promoter sequences.

Primers used for qRT-PCR to validate the RNA-sequencing data

Gene ID	Protein	Sequence(5'-3')	Primer length	Product length	Position
TC010423	CYP450-like protein	TTCAAGAATAAAACCAAAGGCG	22	425bp	127-148
		GTCGTACCCATTGCTGTTTCACA	23		529-551
TC010353	Sensory neuron membrane protein 2	CGTTTCCAGTTGACTTCAAAGTG	23	217bp	182-204
		GTTAGCGGTTTAGATGCCTCGG	22		377-398
TC000517	Pathogenesis related protein 5	AACCGGGCAAACGCGTTT	18	255bp	152-169
		CTCCCAGCGGCTCGATAG	18		389-406
TC013464	Retinaldehyde-binding protein 1	GGAACTTTTGAAAAATGACGACA	23	183bp	117-139
		GCTTGTTTTTCATCTTCTGGCAT	23		277-299
TC009624	Chitinase 8	GATGCTTCCATTCGAGGGT	19	266bp	720-738
		TTTCCCTTGTACATGTAGGGCG	22		964-985
TC014152	Glycine N-methyltransferase	CGCTTGTGGAACTGGAATTG	20	162bp	198-217
		CAATTGGCTTCTCTGATAATCCAAT	25		335-359
TC015780	Serine protease P166	GTTTGGTTGTGCTCCTCCTC	20	273bp	8-27
		TAACTCCTTGGGTGCAGTGA	20		261-280
TC016415	Z9 acyl-CoA desaturase B	CATTTGGCCGCTTTGTATG	19	191bp	148-166
		GCCAAAGTGTTGCAGAAAGTGAG	23		316-338
TC016350	Uncharacterized protein	CTTGTGGAGATTCGACTGGA	20	322bp	105-124
		GACTGGATCGGCGTAGTTG	19		408-426
TC007154	DNA-binding protein D-ETS-6	TGATTTCGAGACCAAAGCAG	20	234bp	822-841
		TCCAAGAGAAACTGCCACAG	20		1036-1055

TC011675	Superoxide dismutase [Cu-Zn]	CTACCAAAGCGACCTCTACGA	21	338bp	156-176
		CTTCCTCCACTTCTATGTTCCCC	23		471-493
TC008096	Dorsal2	CGAACTCAAGGAGAGTCTCAATG	23	342bp	327-349
		GTCTGACCATTTGCCGTTTCG	21		648-668
TC013917	Imaginal disc growth factor 4	GTACTTCAGGGACGGTCAGG	20	231bp	87-106
		ATATCTTCACCTCCGCCAAC	20		298-317
TC003507	CPR18 adult-specific cuticular protein ACP-22	AGTGCACCACGAAGAACAAA	20	207bp	270-289
		ACGAATCCTCGCTTCTTGTC	20		457-476
TC015481	Chitinase 7	TTTGCACGAAGACAACACAA	20	287bp	2427-2446
		AGATCGACGTTTCGTAAGGG	20		2694-2713
TC014634	Chitin synthase A	GGGAGAAGATCGTTGGTTGT	20	284bp	2511-2530
		CCAACATGAGGAAGATGGTG	20		2775-2794
TC011601	Uncharacterized protein	GTCCTGGGTTATCCTTCTTGC	21	146bp	36-56
		CGGAGGCGTAAACAACGTCTC	21		161-181
TC012734	Chitinase 10	AGCTACCACTTGTCGTGTGC	20	266bp	1747-1766
		TACACCACGTGTGTGCAAAG	20		1993-2012
TC005905	Carboxypeptidase B	ATCCGACAGACATTGAGCTG	20	257bp	338-357
		TGTTGCTGGTGAGATCCATT	20		575-594
TC008369	Uncharacterized protein	TCAAGTTTTGCTACCGCTGTG	21	217bp	7-27
		TGATAGCGTTCGGCTTTTCG	20		204-223
TC001770	Chitinase 5	ACTTTGTGGAGGAGCTGAGAC	21	270bp	451-471
		CGACCCAAAGTTGCAAACCA	20		701-720
TC012760	Facilitated trehalose transporter Tret1	CAGCTCAAGTACCCCAACGA	20	137bp	214-233
		GCTAATTTTCTGCCGCAACG	20		331-350
TC015549	Glycine-rich cell wall structural protein	GTTCTTTTCGGCTTTTCGCA	20	104bp	6-25
		CTCCGTAGGAGCTAGATGGG	20		90-109
