# Supplementary material for: A new neuropeptide insect parathyroid hormone iPTH in the red flour beetle Tribolium castaneum
Source: PLoS Genet. 2020 May 4;16(5):e1008772. doi: 10.1371/journal.pgen.1008772 (PMC7224569; doi:10.1371/journal.pgen.1008772)
Supplement: S1 Dataset — (PDF) [file pgen.1008772.s012.pdf]

Dataset_S1. The genes down-regulated in RNAseq of the RNAi samples for Tc-iPTHR1 and Tc-iPTHR2.

GeneID	Gene name - blast	log2(dsiPTHR1/C)	log2(dsiPTHR2/C)2	C-RPKM	dsiPTHR1-RPKM	dsiPTHR2-RPKM
**Extracellular structural components including cuticular proteins**						
TC000369	Larval/pupal cuticle protein H1C	-16.81	-7.78	114.94	0.00	0.52
TC003055	Cuticle protein 65	-16.50	-6.45	92.76	0.00	1.06
TC001177	Larval/pupal cuticle protein H1C-like Protein	-16.29	-6.36	80.35	0.00	0.98
TC003599	Pupal cuticle protein C1B	-14.94	-14.94	31.40	0.00	0.00
TC013817	Pupal cuticle protein Edg-84A-like Protein	-14.52	-5.15	23.47	0.00	0.66
TC003834	Pupal cuticle protein Edg-78E-like Protein	-14.35	-14.35	20.84	0.00	0.00
TC013810	larval cuticle protein 1 [Tribolium castaneum]	-14.23	-14.23	19.16	0.00	0.00
TC003056	cuticle protein 70, isoforms A and B	-14.21	-4.55	19.01	0.00	0.81
TC013816	Adult-specific cuticular protein ACP-20-like Protein	-12.75	-12.75	6.90	0.00	0.00
TC002841	Pupal cuticle protein C1B	-9.95	-9.32	1379.45	1.39	2.16
TC007402	larval cuticle protein F1 [Tribolium castaneum]	-9.05	-18.33	328.47	0.62	0.00
TC000724	Larval cuticle protein A3A-like Protein	-8.79	-7.10	5831.66	13.21	42.55
TC003835	Pupal cuticle protein Edg-78E-like Protein	-8.73	-7.93	437.17	1.03	1.79
TC015549	glycine-rich cell wall structural protein isoform X2 [Tribolium castaneum]	-8.67	-6.45	253.86	0.63	2.90
TC001875	putative glycine-rich cell wall structural protein 1 [Tribolium castaneum]	-8.55	-8.34	294.37	0.78	0.91
TC008769	larval cuticle protein A3A-like [Nicrophorus vespilloides]	-8.34	-6.22	443.87	1.37	5.95
TC000722	Pupal cuticle protein Edg-84A-like Protein	-8.31	-7.43	2322.44	7.33	13.47
TC003830	pupal cuticle protein 20	-8.25	-5.23	83.57	0.27	2.23
TC006262	Pupal cuticle protein C1B-like Protein	-8.18	-18.43	352.66	1.21	0.00
TC000723	Pupal cuticle protein Edg-84A-like Protein	-8.18	-7.08	5580.47	19.21	41.18
TC011925	cuticle protein 16.5, isoform A	-8.15	-7.48	4830.31	16.98	26.98
TC003384	cuticle protein 16.5, isoform B	-7.70	-7.33	1907.69	9.16	11.82
TC000725	Pupal cuticle protein Edg-84A-like Protein	-7.54	-6.51	5759.76	31.00	63.39
TC003385	cuticle protein 16.5, isoform B	-7.14	-5.11	303.14	2.16	8.76
TC003832	Endocuticle structural glycoprotein SgAbd-2-like Protein	-6.62	-6.05	1155.77	11.71	17.49
TC013826	Pupal cuticle protein Edg-84A-like Protein [Tribolium castaneum]	-6.33	-5.12	32.98	0.41	0.95
TC007306	cuticle protein 64 [Tribolium castaneum]	-5.80	-2.97	874.51	15.68	111.49
TC003831	pupal cuticle protein 20 [Tribolium castaneum]	-5.69	-5.15	56.74	1.10	1.60
TC002669	Cuticlin-1 [Habropoda laboriosa]	-5.66	-4.63	31.51	0.63	1.27
TC000163	Cuticlin-1 [Cyphomyrmex costatus]	-5.49	-6.95	188.51	4.20	1.52
TC006646	cuticle protein 18.7-like	-5.37	-5.15	15.12	0.37	0.43
TC003363	Pro-resilin-like Protein	-5.26	-3.83	50.55	1.31	3.56
TC000639	pro-resilin	-5.26	-5.53	157.28	4.11	3.41
TC004546	Cuticle protein 3-like Protein	-4.78	-3.83	2314.44	84.13	162.82

TC000719	Larval cuticle protein A1A-like Protein	-4.71	-4.75	555.89	21.20	20.68
TC000721	Pupal cuticle protein Edg-84A-like Protein	-4.66	-13.80	14.23	0.56	0.00
TC003376	cuticle protein 65 [Tribolium castaneum]	-4.20	-3.99	50.17	2.72	3.16
TC010054	cuticle protein 21-like [Aethina tumida]	-3.87	-2.40	1657.05	113.07	314.97
TC008770	Pupal cuticle protein Edg-84A-like Protein	-3.85	-4.96	52.49	3.64	1.69
TC000980	Cuticular protein hypothetical 33 precursor [Bombyx mori]	-3.79	-3.57	27.10	1.96	2.28
TC001440	Cuticlin-1 [Trachymyrmex zeteki]	-3.61	-3.91	18.24	1.50	1.22
TC007275	glycine-rich cell wall structural protein 1.8-like [Aedes aegypti]	-3.48	-3.40	47.15	4.23	4.47
TC014497	Larval cuticle protein 8-like Protein	-3.35	-2.48	443.89	43.66	79.53
TC004547	Endocuticle structural glycoprotein SgAbd-2-like Protein	-3.06	-2.18	34.81	4.16	7.66
TC014104	Cuticlin-1-like Protein	-2.69	-4.32	124.79	19.40	6.26
TC013134	Larval cuticle protein LCP-30-like Protein	-2.63	-1.07	43.53	7.05	20.70
TC006981	proline-rich extensin-like protein EPR1	-2.55	-1.30	111.31	19.07	45.32
TC004548	endocuticle structural glycoprotein SgAbd-1	-2.50	-2.11	122.76	21.74	28.47
TC003770	Cuticlin-1 [Atta colombica]	-2.23	-2.86	13.47	2.87	1.85
TC013137	Pupal cuticle protein-like Protein	-2.12	-1.04	704.87	161.71	342.89
TC003060	cuticle protein 19.8	-2.06	-1.46	699.99	167.64	254.23
TC009263	cuticular protein analogous to peritrophins 1-D precursor	-1.84	-1.74	56.63	15.81	16.94
TC013812	Pupal cuticle protein Edg-84A-like Protein	-1.73	-1.63	23.24	7.01	7.51
TC015720	larval cuticle protein A3A [Ceratitis capitata]	-1.69	-3.81	156.76	48.68	11.16
TC011140	cuticular protein analogous to peritrophins 3-A1 precursor	-1.55	-3.32	290.15	99.30	29.05
TC004733	cuticular protein analogous to peritrophins 1-A	-1.38	-1.06	78.06	30.07	37.51
TC003048	cuticle protein 19.8	-1.30	-1.30	5351.21	2168.35	2175.96
TC002343	glycine-rich protein DOT1	-12.42	-12.42	5.47	0.00	0.00
TC008858	Agrin-like Protein	-4.44	-5.32	58.90	2.72	1.47
TC014761	cell wall protein RBR3 isoform X2 [Drosophila takahashii]	-3.08	-2.02	201.45	23.87	49.64
TC007273	elastin	-2.03	-1.83	62.87	15.44	17.63
TC007080	cell wall protein RBR3 [Bombus terrestris]	-4.49	-4.64	28.24	1.25	1.13
TC012301	Protein Skeletor, isoforms B/C-like Protein	-2.41	-3.55	27.71	5.22	2.37
TC012207	prisilkin-39	-1.47	-7.89	3251.35	1170.12	13.72
TC011659	keratin, type I cytoskeletal 10-like [Monomorium pharaonis]	-18.75	-18.75	441.77	0.00	0.00
TC011660	keratin, type II cytoskeletal 1-like [Anoplophora glabripennis]	-12.51	-12.51	5.85	0.00	0.00
TC015460	keratin, type I cytoskeletal 9	-6.80	-5.69	419.34	3.77	8.13
TC015245	mucin-12-like [Monomorium pharaonis]	-5.66	-6.84	776.72	15.33	6.80
TC011888	mucin-5AC [Bactrocera oleae]	-5.63	-4.80	602.36	12.16	21.65
TC011601	mucin-3A isoform X2 [Anoplophora glabripennis]	-4.37	-3.98	65.48	3.17	4.14
TC009861	mucin-5AC isoform X1 [Tribolium castaneum]	-4.28	-5.06	46.64	2.40	1.40

TC007806	mucin-5AC-like [Nilaparvata lugens]	-3.87	-4.31	120.72	8.23	6.08
**Cuticle metabolism**						
TC001770	putative chitinase 3-like Protein	-7.76	-9.75	856.64	3.95	1.00
TC011222	Chorion peroxidase-like Protein	-2.41	-2.04	14.54	2.74	3.53
TC015234	Chorion peroxidase-like Protein	-5.08	-4.91	97.69	2.89	3.26
TC012734	putative chitinase 3-like Protein (Chitinase 10)	-3.20	-5.75	258.05	28.02	4.78
TC005652	putative chitinase 3 [Papilio machaon]	-4.76	-3.47	129.72	4.78	11.67
TC010675	truncated knickkopf 3	-4.00	-3.04	179.21	11.18	21.79
TC009636	Alpha-N-acetylgalactosaminidase-like Protein	-1.17	-3.14	41.16	18.27	4.68
TC002496	Tyrosine 3-monooxygenase-like Protein	-2.29	-1.58	435.21	89.11	145.95
**Cyp450 including hormone metabolism**						
TC015295	cytochrome P450 4C1	-3.47	-2.11	6.22	0.56	1.44
TC004159	cytochrome P450 307A1 (spook)	-3.37	-4.15	14.10	1.37	0.79
TC015992	cytochrome P450-like protein	-2.67	-2.68	24.61	3.86	3.84
TC008302	cytochrome P450 301A1	-2.46	-2.17	16.42	2.98	3.66
TC010255	cytochrome P450 6BK2	-2.23	-3.18	7.53	1.61	0.83
TC002722	cytochrome P450-like protein	-1.84	-2.04	42.94	12.01	10.46
**Hormonal signaling pathways**						
TC004812	Ecdysone-induced protein 74EF isoform A isoform X1	-3.11	-3.90	39.85	4.61	2.68
TC004816	Ecdysone-induced protein 74EF isoform B-like Protein	-1.81	-2.20	126.69	36.18	27.66
TC003935	E78 nuclear receptor	-3.17	-3.96	17.92	1.99	1.15
TC008909	hormone receptor in 46-like protein (DHR3)	-3.61	-4.56	132.56	10.89	5.62
TC000543	HR4	-3.90	-4.60	30.47	2.05	1.26
TC015109	Ecdysone-inducible gene E3 [Drosophila melanogaster]	-2.55	-2.87	219.68	37.59	30.09
TC002550	ftz transcription factor 1	-2.11	-1.77	104.99	24.26	30.70
TC011655	Neuropeptide F receptor-like Protein	-3.35	-2.31	13.58	1.33	2.75
TC008163	Ricket, Leucine-rich repeat-containing G-protein coupled receptor 5-like Protein	-2.32	-2.47	21.59	4.31	3.91
TC001181	CG13579-like amine receptor [Tribolium castaneum]	-3.21	-3.36	94.76	10.22	9.23
**Sensory molecular components**						
TC013139	Gustatory receptor for sugar taste 64e-like Protein	-7.11	-5.44	275.40	1.99	6.36
TC003050	Odorant receptor 46a, isoform A-like Protein	-5.88	-8.47	454.28	7.73	1.28
TC008677	chemosensory protein 5	-5.32	-5.69	86.57	2.16	1.68
TC008680	chemosensory protein 9	-5.03	-4.93	863.59	26.48	28.26
TC008676	chemosensory protein 4	-4.75	-4.54	79.02	2.93	3.40
TC008678	chemosensory protein 2	-2.95	-3.74	44.64	5.77	3.35
**Others**						
TC006510	cGMP-dependent protein kinase, isozyme 2 forms cD4/T1/T3A/T3B-like Protein	-1.91	-2.28	33.68	8.98	6.95

TC006392	cys-loop ligand-gated ion channel subunit	-3.03	-1.47	14.68	1.79	5.30
TC015583	Alpha-tocopherol transfer protein-like	-3.15	-2.56	52.81	5.93	8.95
TC005432	cathepsin B precursor	-4.08	-4.86	38.53	2.28	1.33
TC005234	pollen-specific leucine-rich repeat extensin-like protein 2 [Anoplophora glabripennis]	-18.59	-9.02	395.09	0.00	0.76
TC010790	——	-16.17	-5.47	73.81	0.00	1.66
TC008657	serine protease P84	-16.04	-16.04	67.20	0.00	0.00
TC014645	neuropeptide-like protein 31 isoform X1 [Tribolium castaneum]	-15.94	-5.50	63.08	0.00	1.40
TC005904	carboxyopeptidase A	-15.94	-6.93	62.65	0.00	0.51
TC015712	Glucose dehydrogenase [FAD, quinone]-like Protein	-15.19	-6.19	37.38	0.00	0.51
TC001417	Lipase member H-A-like Protein	-14.73	-14.73	27.24	0.00	0.00
TC015789	——	-14.52	-14.52	23.49	0.00	0.00
TC000428	——	-14.42	-4.50	21.86	0.00	0.97
TC010803	serine/threonine-protein kinase pakD-like [Polistes dominula]	-14.35	-14.35	20.92	0.00	0.00
TC015395	Acyl-CoA desaturase-like Protein	-14.17	-14.17	18.48	0.00	0.00
TC015791	——	-14.11	-14.11	17.72	0.00	0.00
TC015790	——	-14.06	-14.06	17.04	0.00	0.00
TC015794	nicotinate phosphoribosyltransferase [Oceanobacillus]	-13.94	-13.94	15.68	0.00	0.00
TC009465	——	-13.81	-1.76	14.37	0.00	4.24
TC006563	——	-13.79	-13.79	14.19	0.00	0.00
TC012151	——	-13.78	-13.78	14.10	0.00	0.00
TC010191	sodium/potassium/calcium exchanger 1-like [Anoplophora glabripennis]	-13.61	-13.61	12.51	0.00	0.00
TC009479	——	-13.52	-13.52	11.75	0.00	0.00
TC012150	Lipase member H-A-like Protein	-13.41	-3.66	10.91	0.00	0.86
TC011665	putative lysozyme-like protein [Dendroctonus ponderosae]	-13.20	-13.20	9.38	0.00	0.00
TC010841	——	-13.19	-4.50	9.36	0.00	0.41
TC003707	zinc finger protein 396	-13.04	-3.69	8.43	0.00	0.65
TC007272	protein TonB-like	-12.82	-12.82	7.22	0.00	0.00
TC008554	Serine proteinase stubble [Papilio machaon]	-12.77	-12.77	7.00	0.00	0.00
TC008104	——	-12.73	-12.73	6.78	0.00	0.00
TC009862	——	-11.96	-11.96	3.98	0.00	0.00
TC001121	tyrosine-protein phosphatase non-receptor type 23-like [Aethina tumida]	-11.80	-3.55	3.57	0.00	0.30
TC012620	——	-10.29	-9.07	748.86	0.60	1.39
TC005225	Zinc metalloprotease zmpB [Apis cerana cerana]	-10.24	-12.02	2413.34	2.00	0.58
TC015556	splicing factor 3A subunit 2	-9.50	-8.28	265.95	0.37	0.85
TC010813	Apolipoprotein D-like Protein	-9.47	-18.10	281.09	0.40	0.00
TC014264	——	-8.56	-17.64	204.05	0.54	0.00
TC010840	nuclear polyadenylated RNA-binding protein 3 [Tribolium castaneum]	-8.34	-6.12	50.48	0.16	0.72

TC012760	Facilitated trehalose transporter Tret1-2 homolog-like Protein	-8.14	-6.92	50.42	0.18	0.42
TC003057	neuropeptide-like precursor 3	-8.05	-7.33	7596.91	28.67	47.10
TC011237	——	-8.03	-6.08	1138.37	4.36	16.88
TC030071-GA	——	-8.02	-6.80	56.17	0.22	0.50
TC005613	surface protein isoform X1	-7.60	-6.39	111.74	0.57	1.33
TC008369	——	-7.48	-7.85	448.82	2.51	1.95
TC012679	serine, glycine and glutamine-rich protein-like [Anoplophora glabripennis]	-7.40	-9.50	299.28	1.78	0.41
TC005503	Vitellogenin-2-like Protein	-7.39	-7.76	138.66	0.83	0.64
TC006612	Signal recognition particle subunit SRP68-like Protein	-7.20	-5.40	29.38	0.20	0.70
TC005905	carboxyopeptidase A	-7.03	-7.49	674.38	5.17	3.75
TC010421	RNA polymerase II degradation factor 1	-6.98	-9.70	962.02	7.62	1.15
TC009528	——	-6.89	-6.99	28.49	0.24	0.22
TC000548	serine protease-like protein	-6.88	-15.15	36.44	0.31	0.00
TC006093	stress response protein [Lasius niger]	-6.87	-6.43	258.70	2.21	2.99
TC011658	——	-6.63	-6.42	32.87	0.33	0.38
TC010196	filaggrin-2-like isoform X10 [Priapulus caudatus]	-6.59	-7.96	59.45	0.62	0.24
TC015593	alpha/beta hydrolase [Myxococcus virescens]	-6.56	-6.93	252.64	2.68	2.08
TC008235	altered inheritance of mitochondria protein 3	-6.51	-4.71	25.25	0.28	0.97
TC008659	serine proteinase stubble [Tribolium castaneum]	-6.48	-7.27	50.12	0.56	0.32
TC005614	surface protein isoform X1	-6.38	-6.75	83.09	1.00	0.77
TC009889	high mobility group nucleosome-binding domain-containing protein 5 isoform X1 [Bactrocera latifrons]	-6.36	-6.14	52.37	0.64	0.74
TC010419	C-type lectin precursor	-6.22	-5.89	375.07	5.04	6.34
TC002385	——	-6.14	-5.92	42.38	0.60	0.70
TC016312	myb-like protein Q isoform X1 [Bactrocera dorsalis]	-6.03	-15.90	61.31	0.94	0.00
TC008392	——	-5.87	-6.24	26.59	0.45	0.35
TC001023	serine protease P22	-5.86	-3.32	16.38	0.28	1.64
TC016368	invected	-5.66	-6.44	157.39	3.11	1.81
TC006985	alpha-protein kinase 1 isoform X1	-5.63	-4.16	109.23	2.21	6.10
TC015724	glucose dehydrogenase [FAD, quinone]	-5.61	-4.50	50.48	1.03	2.23
TC012680	serine, glycine and glutamine-rich protein-like [Anoplophora glabripennis]	-5.53	-7.31	64.83	1.40	0.41
TC013560	juvenile hormone binding protein-like precursor [Aphis gossypii]	-5.50	-5.06	160.14	3.55	4.80
TC006726	spatzle 4	-5.49	-5.28	12.31	0.27	0.32
TC015195	Protein takeout-like Protein	-5.27	-5.47	56.46	1.46	1.27
TC010192	tetra-peptide repeat homeobox protein 1-like [Nicrophorus vespilloides]	-5.17	-6.54	94.03	2.60	1.01
TC004622	serine protease H51	-5.14	-4.72	57.89	1.64	2.19
TC012819	putative fatty acyl-CoA reductase CG5065	-5.12	-5.65	27.64	0.79	0.55
TC006122	——	-5.10	-16.35	83.27	2.42	0.00

TC003763	——	-5.04	-5.03	65.54	2.00	2.01
TC014638	——	-5.02	-6.12	125.03	3.87	1.80
TC012826	——	-4.81	-5.28	161.04	5.73	4.16
TC016313	RNA polymerase II degradation factor 1 isoform X1 [Aedes aegypti]	-4.72	-5.85	574.69	21.73	9.98
TC002085	serpin peptidase inhibitor 2	-4.72	-4.50	36.89	1.40	1.63
TC010842	Replicase polyprotein 1a [Dufourea novaeangliae]	-4.71	-13.30	10.10	0.38	0.00
TC005375	Larval serum protein 2-like Protein	-4.69	-3.80	90.91	3.52	6.52
TC008844	la-related protein 6	-4.59	-4.71	658.54	27.26	25.14
TC007264	lipase 3-like	-4.59	-5.96	17.75	0.74	0.29
TC006658	alkaline phosphatase	-4.40	-4.18	36.46	1.73	2.01
TC014301	leucine-rich repeat and fibronectin type-III domain-containing protein 5	-4.39	-3.85	54.39	2.59	3.76
TC012827	phosphotidylinositol phosphatase PTPRQ-like [Tropilaelaps mercedesae]	-4.26	-5.05	364.29	18.95	11.00
TC002293	pipe	-4.24	-4.89	57.35	3.04	1.93
TC002945	titin isoform X1 [Tribolium castaneum]	-4.23	-3.99	86.46	4.60	5.43
TC007763	zinc finger protein 512B [Tribolium castaneum]	-4.19	-3.03	125.86	6.91	15.39
TC015158	Dehydrogenase/reductase SDR family protein 7-like	-4.06	-3.95	74.00	4.44	4.78
TC015721	Glucose dehydrogenase [FAD, quinone]-like Protein	-4.02	-5.15	78.59	4.84	2.22
TC014619	——	-4.02	-1.80	52.91	3.27	15.20
TC006564	——	-3.94	-4.57	20.30	1.33	0.86
TC001783	RNA polymerase II degradation factor 1 [Bombus impatiens]	-3.92	-4.51	102.70	6.79	4.50
TC007252	MD-2-related lipid-recognition protein-like Protein	-3.86	-3.65	16.85	1.16	1.35
TC014741	Blimp-1	-3.82	-4.13	63.66	4.51	3.64
TC013505	——	-3.77	-5.14	94.29	6.91	2.67
TC015554	Dehydrogenase/reductase SDR family protein 7-like	-3.76	-3.89	344.90	25.41	23.27
TC011602	——	-3.76	-4.13	188.09	13.91	10.77
TC011657	——	-3.75	-3.53	11.54	0.86	1.00
TC012466	seminal fluid protein [Leptinotarsa decemlineata]	-3.68	-4.29	171.78	13.37	8.81
TC002984	sushi, von Willebrand factor type A, EGF and pentraxin domain-containing protein 1 [Acyrthosiphon pisum]	-3.67	-3.38	24.44	1.92	2.35
TC001977	Xanthine dehydrogenase-like Protein	-3.61	-4.66	21.27	1.74	0.84
TC006224	——	-3.56	-5.11	229.19	19.46	6.61
TC011736	WW domain-containing oxidoreductase-like Protein	-3.42	-4.62	48.96	4.57	1.99
TC007085	——	-3.41	-2.34	206.46	19.38	40.91
TC014646	T-complex-associated testis-expressed protein 1-like Protein	-3.37	-4.15	3.78	0.37	0.21
TC009212	EGF-like domain-containing protein 2 [Tribolium castaneum]	-3.28	-2.61	56.97	5.86	9.31
TC005065	serpin peptidase inhibitor 6	-3.27	-3.79	23.25	2.41	1.68
TC013617	heat shock protein DDB_G0288861	-3.26	-2.73	5.23	0.54	0.79
TC002341	Dorsal-ventral patterning protein tolloid-like Protein	-3.20	-6.38	58.78	6.39	0.71

TC012467	Sulfotransferase family cytosolic 1B member 1-like Protein	-3.10	-1.47	14.67	1.71	5.29
TC015830	Putative phosphatidate phosphatase-like Protein	-3.04	-4.15	14.59	1.77	0.82
TC007042	Vitellogenin-3-like Protein	-3.03	-3.13	20.20	2.48	2.30
TC010879	WW domain-containing oxidoreductase-like Protein	-3.02	-2.81	7.20	0.89	1.03
TC013657	circadian clock-controlled protein-like [Megachile rotundata]	-3.00	-1.63	2216.49	277.77	715.02
TC013416	tripartite motif-containing protein 45	-2.97	-1.46	22.45	2.86	8.16
TC005627	ankycorbin isoform X1	-2.96	-3.99	14.56	1.87	0.92
TC013283	NADPH oxidase 5 isoform X1 [Tribolium castaneum]	-2.93	-2.50	34.70	4.54	6.14
TC013787	Zinc finger protein 570-like Protein	-2.89	-2.34	18.96	2.55	3.75
TC006664	pollen-specific leucine-rich repeat extensin-like protein 1	-2.89	-4.67	8439.91	1140.25	331.27
TC004654	serine protease P54	-2.84	-3.08	21.61	3.01	2.56
TC008120	——	-2.83	-2.94	19.54	2.74	2.55
TC015337	Fatty acid synthase-like Protein	-2.79	-4.13	68.68	9.94	3.93
TC013042	serine protease P133	-2.79	-2.22	16.25	2.35	3.48
TC008236	probable basic-leucine zipper transcription factor I	-2.76	-3.86	10.41	1.54	0.72
TC009126	heat shock protein 68b	-2.67	-2.46	4.52	0.71	0.82
TC005192	DNA replication licensing factor Mcm2-like Protein	-2.66	-4.45	10.59	1.67	0.49
TC013568	Protein scarlet-like Protein	-2.64	-1.95	28.69	4.60	7.43
TC004635	serine protease P53	-2.62	-4.94	563.36	91.42	18.33
TC000393	Synaptic vesicle glycoprotein 2B-like Protein	-2.56	-2.65	165.85	28.08	26.49
TC013659	Protein takeout-like Protein	-2.56	-2.23	614.26	104.08	131.21
TC011656	osiris 24-like protein [Lasius niger]	-2.56	-2.34	36.08	6.12	7.11
TC009318	Protein Wnt-5-like Protein	-2.55	-12.49	5.74	0.98	0.00
TC004557	aminopeptidase N-like protein	-2.55	-3.16	297.25	50.88	33.18
TC000547	serine protease P13	-2.54	-4.50	180.17	31.03	7.97
TC004298	——	-2.53	-4.12	41.73	7.25	2.40
TC009716	——	-2.52	-2.05	25.09	4.36	6.08
TC003708	C-type lectin precursor	-2.48	-2.26	572.73	102.73	119.30
TC016319	SGNH/GDSL hydrolase family protein	-2.47	-4.07	54.01	9.72	3.22
TC003194	scabrous	-2.43	-2.80	15.52	2.89	2.23
TC015490	putative eukaryotic translation initiation factor 3 theta subunit	-2.39	-1.06	43.07	8.20	20.64
TC009833	Putative fatty acyl-CoA reductase CG5065-like Protein	-2.39	-3.67	223.66	42.70	17.60
TC008206	myrosinase-binding protein 2	-2.37	-3.15	12.77	2.48	1.44
TC008910	——	-2.37	-2.69	56.88	11.03	8.83
TC006120	Putative fatty acyl-CoA reductase CG5065-like Protein	-2.35	-2.97	94.99	18.66	12.13
TC014184	rhoGEF domain-containing protein gxcl isoform X1	-2.34	-4.36	1026.46	202.07	50.06
TC002943	Zinc transporter ZIP1-like Protein	-2.34	-1.70	49.53	9.76	15.20

TC015589	——	-2.30	-2.67	27.36	5.54	4.29
TC005793	Dehydrogenase/reductase SDR family protein 7-like	-2.30	-3.08	11.00	2.23	1.30
TC011214	Putative fatty acyl-CoA reductase CG5065-like Protein	-2.30	-1.86	64.55	13.14	17.84
TC010771	putative E3 ubiquitin-protein ligase sinah	-2.28	-2.28	20.19	4.16	4.17
TC012794	——	-2.27	-3.32	24.50	5.07	2.45
TC003396	——	-2.23	-3.82	7.01	1.49	0.50
TC015399	Fatty acid synthase-like Protein	-2.22	-5.84	73.15	15.66	1.28
TC009368	Rhophilin-2-like Protein	-2.20	-1.33	22.12	4.83	8.81
TC007331	GILT-like protein F37H8.5	-2.18	-2.38	77.82	17.20	14.98
TC012390	serine protease H129	-2.17	-2.40	55.12	12.29	10.45
TC008455	putative mediator of RNA polymerase II transcription subunit 26	-2.16	-2.31	9.98	2.23	2.01
TC011335	——	-2.15	-1.14	12.32	2.77	5.60
TC010098	——	-2.15	-1.54	22.34	5.03	7.69
TC011379	tartan/capricious-like protein	-2.14	-1.55	114.95	26.15	39.21
TC013059	Putative ferric-chelate reductase 1 homolog-like Protein	-2.13	-1.39	1108.95	253.36	422.80
TC010154	skin secretory protein xP2	-2.12	-2.08	152.64	35.12	36.10
TC015723	Glucose dehydrogenase	-2.11	-2.96	168.10	38.91	21.58
TC006428	Monocarboxylate transporter 7-like Protein	-2.10	-2.52	10.65	2.48	1.86
TC002263	——	-2.09	-1.43	54.02	12.67	20.07
TC003987	SEC14 domain and spectrin repeat-containing protein 1-like ProteinSEC14???????????????????1???	-2.09	-2.18	56.22	13.24	12.37
TC000611	Alpha-tocopherol transfer protein-like	-2.05	-2.14	27.26	6.57	6.17
TC008809	integrase core domain-containing protein	-2.05	-2.65	5.57	1.34	0.89
TC016278	Elongation of very long chain fatty acids protein AAEL008004-like Protein	-2.05	-2.68	38.28	9.26	5.98
TC001129	cadherin 23	-2.04	-1.31	4.67	1.13	1.89
TC009706	heat shock protein 68a	-2.02	-2.65	4.47	1.10	0.71
TC003081	serine protease H42	-1.99	-2.35	47.56	11.94	9.36
TC003047	neuropeptide-like 3 isoform X2	-1.99	-2.64	1418.72	356.48	228.13
TC008099	protein ovo isoform X2	-1.97	-2.39	103.39	26.47	19.77
TC015152	Arylsulfatase B-like Protein	-1.94	-2.63	66.70	17.38	10.75
TC010578	Putative fatty acyl-CoA reductase CG5065-like Protein	-1.93	-2.33	56.69	14.84	11.28
TC006736	Eukaryotic translation initiation factor 3 subunit I-like Protein	-1.87	-2.06	213.94	58.65	51.24
TC010791	Deoxynucleoside kinase-like Protein	-1.85	-2.17	18.35	5.09	4.09
TC012577	Histone H3-like Protein	-1.84	-2.08	28.63	8.00	6.75
TC012621	26S proteasome non-ATPase regulatory subunit 11-like Protein	-1.83	-2.53	595.64	167.09	103.17
TC009480	histidine-rich glycoprotein	-1.81	-4.57	1132.49	322.03	47.70
TC009808	putative beta-hexosaminidase fdl-like Protein	-1.78	-2.37	213.65	62.39	41.20
TC015693	Sarcoplasmic calcium-binding protein-like Protein	-1.71	-2.30	40.61	12.43	8.25

TC002785	serine protease P40	-1.70	-3.41	21.80	6.69	2.05
TC006471	Very long-chain-fatty-acid--CoA ligase bubblegum-like Protein	-1.70	-1.75	107.30	32.99	31.82
TC005701	Protein white-like Protein	-1.61	-2.05	38.42	12.59	9.26
TC011052	Angiotensin-converting enzyme-like Protein	-1.54	-2.31	1014.06	347.79	204.06
TC007265	altered inheritance of mitochondria protein 3-like [Aethina tumida]	-1.52	-2.26	56.87	19.81	11.86
TC001909	——	-1.51	-2.09	19.22	6.75	4.52
TC010028	A disintegrin and metalloproteinase with thrombospondin motifs 7-like	-1.45	-2.34	18.10	6.64	3.59
TC000601	Venom allergen 3-like Protein	-1.44	-2.01	583.69	214.86	145.36
TC012152	Lipase member H-A-like Protein	-1.44	-2.53	13.88	5.12	2.41
TC002709	Apoptosis 2 inhibitor-like Protein	-1.44	-2.22	31.23	11.53	6.69
TC011635	Lipase 1-like Protein	-1.42	-2.43	41.34	15.43	7.68
TC010429	Protein O-mannosyltransferase 1-like Protein	-1.41	-2.30	51.87	19.53	10.50
TC014544	G2/mitotic-specific cyclin-B-like Protein	-1.39	-2.91	12.08	4.60	1.60
TC005411	Beta-glucuronidase-like Protein	-1.36	-2.37	61.06	23.76	11.80
TC013108	Villin-like protein quail	-1.34	-2.49	11.34	4.47	2.02
TC005142	putative RNA-directed DNA polymerase from transposon BS-like Protein	-1.33	-2.31	17.46	6.93	3.52
TC010547	Myosin-VIIa-like Protein	-1.31	-2.08	31.72	12.79	7.48
TC014023	polo	-1.29	-2.86	14.94	6.12	2.06
TC000269	BTB/POZ domain-containing protein kctd15-like Protein	-1.27	-2.06	17.21	7.12	4.13
TC013030	Neprilysin-2-like Protein	-1.27	-2.36	273.80	113.24	53.37
TC013713	DNA replication licensing factor Mcm7-like Protein	-1.25	-2.04	30.30	12.72	7.39
TC013090	myosin-9 isoform X2	-1.23	-2.14	192.46	82.00	43.69
TC016058	trithorax group protein osa-like [Anoplophora glabripennis]	-1.20	-2.52	12.74	5.55	2.22
TC007059	Ankyrin repeat and BTB/POZ domain-containing protein 2-like Protein	-1.04	-2.17	9.05	4.39	2.01
