# Supplementary material for: A new neuropeptide insect parathyroid hormone iPTH in the red flour beetle Tribolium castaneum
Source: PLoS Genet. 2020 May 4;16(5):e1008772. doi: 10.1371/journal.pgen.1008772 (PMC7224569; doi:10.1371/journal.pgen.1008772)
Supplement: S2 Dataset — (PDF) [file pgen.1008772.s013.pdf]

Dataset_S2. The genes up-regulated in RNAseq of the RNAi samples for Tc-iPTHR1 and Tc- iPTHR2.

GeneID	Gene name - blast	log2(dsiPTHR1/C)	log2(dsiPTHR2/C)2	C-RPKM	dsiPTHR1-RPKM	dsiPTHR2-RPKM
Extracellular structural components including cuticular proteins						
TC014315	glycine-rich cell wall structural protein [Tribolium castaneum]	1.16	2.02	92.08	205.72	372.31
TC002907	cuticle protein 12.5-like [Folsomia candida]	1.56	2.88	71.20	210.26	523.05
TC012829	cuticular protein precursor	1.71	2.94	106.43	348.78	814.98
TC000720	Pupal cuticle protein Edg-84A-like Protein	1.82	3.15	899.72	3180.12	8010.62
TC008226	cuticle protein 63 [Tribolium castaneum]	1.89	3.03	10.21	37.79	83.31
TC014500	Larval cuticle protein 8-like Protein	1.97	2.80	1307.04	5123.49	9103.95
TC009580	peritrophic matrix protein 2-C precursor	2.12	1.38	3.74	16.24	9.71
TC003041	cuticle protein 64	2.21	3.42	729.30	3365.25	7808.24
TC013136	Larval cuticle protein 8-like Protein	2.23	2.60	1018.49	4777.12	6163.00
TC012828	cuticle protein 18.7	2.50	3.92	121.10	684.75	1834.04
TC003386	cuticle protein 64 [Tribolium castaneum]	2.73	4.15	482.18	3206.15	8564.06
TC009232	peritrophic matrix protein 3 precursor [Tribolium castaneum]	2.79	3.13	30.44	209.89	266.03
TC003274	peritrophic matrix protein 2-A precursor [Tribolium castaneum]	2.79	4.15	6.10	42.28	108.26
TC009873	cuticle protein 7	2.93	4.66	5.24	40.00	132.84
TC009957	anti-diruetic peptide (ADF, cuticle)	2.97	4.49	472.39	3688.76	10638.40
TC009956	ADFb like protein [Tribolium castaneum]	3.47	4.96	100.00	1109.06	3119.04
TC016350	putative chitin binding domain protein [Culex tarsalis]	3.55	3.27	53.33	625.36	514.97
TC008233	cuticle protein 64 isoform X1 [Tribolium castaneum]	3.68	5.06	1.48	18.95	49.52
TC016345	putative chitin binding domain protein	4.09	3.70	2.03	34.69	26.44
TC008889	larval/pupal cuticle protein H1C	4.79	6.02	1.07	29.59	69.37
TC008890	cuticle protein 21	5.22	6.83	0.80	29.69	90.62
TC016346	putative chitin binding domain protein	5.99	5.52	4.88	311.28	224.34
TC013671	adult cuticle protein 1-like	6.80	8.11	7.47	830.41	2058.11
TC016349	putative chitin-binding protein [Lutzomyia longipalpis]	1.86	2.66	64.21	233.37	405.74
TC013827	Adult-specific cuticular protein ACP-20-like Protein	1.78	2.36	20.87	71.49	107.37
TC006098	peritrophic matrix protein 14 precursor [Tribolium castaneum]	3.27	3.41	42.67	413.02	454.29
TC016348	putative chitin binding domain protein	5.30	2.44	3.17	124.95	17.16
TC013013	keratin, type I cytoskeletal 9	1.37	2.05	104.31	270.06	431.02
TC003039	keratin-associated protein 19-9b	1.97	3.14	1201.18	4705.49	10576.41
TC003387	keratin-associated protein 19-9b	2.30	3.52	287.60	1415.70	3302.44
TC003037	keratin-associated protein 19-9b [Tribolium castaneum]	3.75	4.61	251.42	3372.70	6121.35
TC005092	keratin-associated protein 19-9b	4.93	5.45	7.53	229.81	330.32
TC004827	keratin, type I cytoskeletal 9	5.25	6.00	18.17	691.94	1162.72
TC005091	prismalin-14	1.55	2.16	85.89	252.31	382.57

TC005098	prismalin-14	1.82	2.49	106.98	377.67	602.79
TC003038	prismalin-14	3.19	4.44	652.45	5947.84	14136.57
TC005097	prismalin-14 [Tribolium castaneum]	3.60	4.32	9.04	109.28	181.02
TC003042	prismalin-14	4.46	6.05	18.74	411.39	1239.74
TC006358	prismalin-14	3.47	4.86	1657.06	18401.39	48235.20
TC003044	prismalin-14	9.80	11.16	3.12	2791.43	7134.81
**Cuticle metabolism**						
TC013662	chitin deacetylase 6 precursor [Tribolium castaneum]	3.12	1.41	4.46	38.77	11.85
TC009624	Chitinase 8	3.16	4.06	57.20	510.73	956.51
TC009176	Chitinase 16	3.22	1.23	10.26	95.89	24.04
TC009177	Chitinase 9	4.37	2.31	3.13	64.89	15.51
TC008126	Chitinase-3-like protein 1	3.79	4.42	1.49	20.65	31.97
**Cyp450**						
TC012503	cytochrome P450-like protein	1.84	2.31	13.79	49.53	68.54
TC006444	cytochrome P450 9Z4	2.09	1.68	42.05	179.59	134.62
TC006441	cytochrome P450 9Z1	2.12	2.06	7.65	33.14	31.83
TC010245	cytochrome P450 6BR3 [Tribolium castaneum]	2.32	1.86	2.24	11.22	8.12
TC015290	cytochrome P450-like protein	2.56	1.89	2.25	13.30	8.36
TC010423	cytochrome P450 CYP4BN1	6.34	2.11	3.92	316.80	16.88
TC008523	cytochrome P450-like protein	3.48	2.41	1.20	13.37	6.38
**Immune functions**						
TC010517	Defensin-like Protein/Defensin 3	3.09	3.75	49.39	419.09	663.51
TC006250	Defensin-like Protein/Defensin 1	3.14	3.86	31.00	273.44	448.69
TC007737	Attacin 2	4.12	5.21	19.14	333.31	708.73
TC007738	Attacin 1	4.17	5.42	24.12	434.81	1033.14
TC013620	Peptidoglycan-recognition protein SC2-like Protein	1.94	2.86	34.99	134.26	253.83
TC010611	Peptidoglycan-recognition protein SA-like Protein	1.55	2.28	29.36	86.00	142.74
**Others**						
TC000804	Acetylcholinesterase-like Protein	2.20	2.57	27.34	125.56	161.77
TC010350	Sensory neuron membrane protein 2-like Protein	2.33	2.28	6.38	32.19	30.91
TC011002	Cathepsin L-like Protein [Tribolium castaneum]	2.58	2.02	4.09	24.43	16.55
TC002952	cathepsin B precursor	3.02	1.28	11.76	95.42	28.49
TC001950	Cathepsin L-like Protein	3.98	2.01	3.13	49.56	12.61
TC011000	cathepsin L precursor	4.10	2.15	21.41	367.53	94.73
TC016303	Senecionine N-oxygenase-like Protein	1.13	2.28	23.63	51.72	114.77
TC008730	probable serine/threonine-protein kinase tsuA	1.23	2.16	38.80	90.80	173.56
TC015614	Homogentisate 1,2-dioxygenase-like Protein	1.40	2.12	31.97	84.20	139.01

TC005493	putative oxidase/peroxidase [Danaus plexippus plexippus]	1.51	2.29	7.79	22.17	38.14
TC001115	DNA-directed RNA polymerase II subunit RPB1-like isoform X2 [Linepithema humile]	1.55	3.69	8.16	23.82	105.31
TC008485	early nodulin-75-like [Anoplophora glabripennis]	1.55	1.67	10.50	30.69	33.50
TC000087	Protein henna-like Protein	1.57	2.35	12.46	36.88	63.32
TC001259	aminopeptidase N-like protein	1.62	2.14	18.26	56.31	80.42
TC008777	putative beta-hexosaminidase fdl-like Protein	1.66	2.27	13.40	42.36	64.41
TC000445	alpha-glucosidase [Anaerobranca gottschalkii]	1.68	2.26	39.37	125.96	189.11
TC030075	Transmembrane protease serine 9-like Protein	1.71	2.31	4.26	13.99	21.11
TC014375	serine protease H148	1.74	2.24	20.59	68.96	97.21
TC010929	serine protease H111	1.79	2.94	5.31	18.39	40.66
TC003045	neuropeptide-like 4	1.81	2.53	308.28	1082.89	1776.07
TC030074	Transmembrane protease serine 9-like Protein	1.86	2.76	8.59	31.25	58.19
TC007601	DNA polymerase beta [Orchesella cincta]	1.87	2.52	929.78	3403.90	5330.53
TC015373	apolipophorin-III	1.90	2.77	1214.00	4523.16	8271.96
TC015840	Protein takeout-like Protein	1.95	2.34	65.43	252.88	330.31
TC012702	4-hydroxyphenylpyruvate dioxygenase-like Protein	1.97	2.44	196.58	768.66	1067.65
TC010937	serine protease H118	2.00	2.52	39.79	159.51	227.94
TC003043	——	2.00	2.68	73.26	293.67	470.86
TC006284	Gamma-glutamyltranspeptidase 1-like Protein	2.01	2.34	5.36	21.59	27.10
TC007930	——	2.04	1.44	11.41	46.99	31.01
TC007689	Fatty acid synthase-like Protein	2.05	2.52	4.83	19.98	27.60
TC004657	Vitellogenin-3-like Protein	2.06	1.25	18.38	76.59	43.76
TC013757	Wilms tumor protein 1-interacting protein homolog [Aethina tumida]	2.06	1.77	2.68	11.21	9.14
TC011471	Acyl-CoA Delta(11) desaturase-like Protein	2.09	3.21	33.97	144.13	313.71
TC015572	inter-alpha-trypsin inhibitor heavy chain H4-like	2.10	1.08	10.62	45.56	22.44
TC003036	neurofilament heavy polypeptide-like [Solanum tuberosum]	2.11	2.77	68.06	293.82	465.64
TC005483	non-ribosomal peptide synthetase [Paenibacillus sp. FSL R7-0273]	2.14	1.02	201.07	884.27	407.05
TC004102	——	2.14	2.12	6.36	27.99	27.58
TC004539	Protein slit-like Protein	2.16	2.56	41.55	185.45	245.32
TC001162	Venom acid phosphatase Acph-1-like Protein	2.16	1.07	34.83	155.73	72.98
TC012551	hemocytin isoform X1	2.17	1.26	3.70	16.65	8.84
TC010911	——	2.19	2.97	4.79	21.79	37.57
TC010927	serine protease H110	2.19	2.97	43.59	198.36	341.12
TC000293	——	2.19	2.26	18.22	83.28	87.49
TC005430	mating-type protein A-alpha Z4-like [Camponotus floridanus]	2.26	1.79	4.29	20.61	14.82
TC016218	Neutral alpha-glucosidase AB-like Protein	2.29	2.18	6.23	30.44	28.19
TC012573	serine protease H130	2.29	2.46	10.33	50.62	56.79

TC010936	serine protease H117	2.30	3.51	7.40	36.37	84.46
TC014068	protein NPC2 homolog isoform X1	2.32	1.76	8.42	41.91	28.51
TC010909	——	2.34	3.93	5.35	27.09	81.54
TC002184	Niemann-Pick C1 protein-like Protein	2.35	1.56	0.90	4.57	2.65
TC005124	——	2.36	3.47	36.43	187.24	404.56
TC000291	——	2.36	1.03	16.38	84.23	33.35
TC000515	Pathogenesis-related protein 5-like Protein	2.37	-1.49	21.03	108.33	7.50
TC016415	Acyl-CoA Delta(11) desaturase-like Protein	2.39	2.75	36.22	189.47	243.66
TC002209	ATP-dependent DNA helicase PIF1 [Folsomia candida]	2.40	2.05	2.45	12.95	10.16
TC013709	serine protease P145	2.42	1.97	171.12	918.86	669.34
TC007845	Cytochrome b5-like Protein	2.45	3.09	5.87	32.10	50.00
TC010940	serine protease P121	2.45	2.15	46.70	255.31	206.68
TC004624	serine protease P52	2.46	2.08	3.88	21.44	16.41
TC006485	Protein msta, isoform A-like Protein	2.49	2.70	1.38	7.71	8.95
TC000222	E-cadherin-like protein	2.49	1.28	5.81	32.66	14.12
TC005602	proline-rich extensin-like protein EPR1	2.50	2.80	213.15	1202.03	1483.92
TC008357	Maltase A1-like Protein	2.50	-1.43	9.30	52.75	3.46
TC012574	serine protease H131	2.52	1.84	12.60	72.51	45.18
TC007017	serine protease P76	2.52	2.73	42.78	246.16	283.39
TC010908	serine protease H106	2.53	3.22	3.27	18.81	30.33
TC003029	phosphotransferase [Oryctes borbonicus]	2.53	2.37	3.51	20.26	18.16
TC015792	——	2.54	-2.29	53.01	308.45	10.80
TC013040	Putative sodium-dependent multivitamin transporter-like Protein	2.55	1.43	6.59	38.56	17.78
TC000175	Peroxidase-like Protein	2.58	3.01	1.02	6.12	8.21
TC015285	Endothelin-converting enzyme 1-like Protein	2.64	1.68	2.72	17.03	8.74
TC008504	serine protease P80	2.66	1.42	17.05	107.69	45.47
TC015579	serine protease P160	2.66	2.21	18.01	113.90	83.27
TC010904	serine protease H101	2.71	1.67	5.15	33.62	16.33
TC010496	Glycine dehydrogenase (decarboxylating), mitochondrial-like Protein	2.71	2.28	3.39	22.16	16.47
TC010932	serine protease H113	2.72	2.51	9.23	60.73	52.40
TC003046	serine/threonine-protein phosphatase 1 regulatory subunit 10 isoform X2 [Athalia rosae]	2.72	3.78	421.34	2780.96	5768.29
TC009362	cathepsin L precursor [Tribolium castaneum]	2.73	6.81	1.30	8.61	145.70
TC008057	Lipid storage droplets surface-binding protein 1-like Protein	2.79	2.49	71.89	497.16	404.88
TC007600	——	2.80	2.50	111.77	776.75	633.87
TC005601	proline-rich extensin-like protein EPR1 isoform X1 [Tribolium castaneum]	2.80	2.98	668.05	4649.20	5258.54
TC010052	myrosinase 1	2.81	2.09	2.73	19.12	11.63
TC011614	Radical S-adenosyl methionine domain-containing protein 2-like Protein	2.89	3.01	10.15	75.23	81.55

TC006659	alkaline phosphatase [Tribolium castaneum]	2.92	1.17	48.57	368.05	109.12
TC013525	DNA-binding response regulator [Demequina aestuarii]	2.93	1.69	8.04	61.41	25.90
TC014391	serine protease P149	3.01	2.53	21.07	169.25	121.40
TC002766	——	3.03	2.85	3.72	30.42	26.88
TC006298	Tetratricopeptide repeat protein 26-like Protein	3.03	2.66	1.40	11.41	8.83
TC010588	E3 ubiquitin-protein ligase sina [Tribolium castaneum]	3.03	2.53	2.35	19.21	13.58
TC007184	Lipase 3-like Protein	3.05	1.58	3.41	28.27	10.22
TC015746	Fasciclin-1-like Protein	3.07	2.06	1.42	11.90	5.92
TC010930	serine protease H112	3.08	2.93	6.86	58.17	52.18
TC004033	——	3.09	2.48	1.42	12.11	7.91
TC014864	——	3.14	3.84	11.13	98.39	159.56
TC012575	serine protease H132	3.20	3.31	1.56	14.27	15.42
TC009954	——	3.25	4.52	3.83	36.44	87.79
TC016352	MD2-like 8 isoform (2) precursor [Tribolium castaneum]	3.26	2.00	4.92	47.26	19.65
TC014091	putative multidrug resistance-associated protein lethal(2)03659-like Protein	3.29	1.37	1.97	19.25	5.09
TC011564	Pathogenesis-related protein 5-like Protein	3.29	2.03	11.50	112.87	47.01
TC011675	Superoxide dismutase [Cu-Zn]-like Protein	3.36	4.32	18.84	192.85	377.44
TC015782	Zinc carboxypeptidase A 1-like Protein	3.38	1.37	7.30	75.82	18.85
TC015580	serine protease P161	3.41	3.34	3.33	35.51	33.81
TC006639	——	3.45	2.85	8.91	97.65	64.23
TC006990	——	3.46	3.97	6.24	68.90	97.74
TC000289	golgin subfamily A member 5-like [Anoplophora glabripennis]	3.51	2.10	36.34	412.61	155.47
TC013464	retinaldehyde-binding protein 1	3.55	2.79	35.07	411.74	241.83
TC015780	serine protease P166	3.89	3.84	44.04	654.22	629.35
TC008506	chondroitin proteoglycan-2 isoform X2	3.95	4.30	0.92	14.21	18.06
TC003053	neuropeptide-like 3 isoform X2	4.17	5.21	17.50	315.53	646.12
TC007073	histidine-rich glycoprotein	4.18	3.95	0.77	13.97	11.93
TC014152	Glycine N-methyltransferase-like Protein	4.29	6.15	7.96	156.21	566.74
TC004216	chondroitin proteoglycan-2 isoform X2	4.33	4.69	1.03	20.60	26.47
TC000292	——	4.34	3.92	2.94	59.50	44.55
TC000288	——	4.51	2.59	27.90	634.57	167.74
TC000517	Pathogenesis-related protein 5-like Protein	4.62	6.43	3.18	78.03	274.03
TC003381	——	4.65	5.98	27.95	702.30	1768.68
TC010935	serine protease H116	4.95	7.06	0.33	10.30	44.53
TC003382	neuropeptide-like precursor 4	5.47	6.95	5.54	245.26	682.85
TC006157	——	7.58	4.70	0.30	57.05	7.73
TC016121	Serine protease 3-like Protein	14.73	12.84	0.00	27.18	7.35
