# Supplementary material for: A new neuropeptide insect parathyroid hormone iPTH in the red flour beetle Tribolium castaneum
Source: PLoS Genet. 2020 May 4;16(5):e1008772. doi: 10.1371/journal.pgen.1008772 (PMC7224569; doi:10.1371/journal.pgen.1008772)
Supplement: Attachment — Submitted filename: review_Xie_plosgenet2019_rev_Mirabeau.docx [file pgen.1008772.s014.docx]

**General comments:**

I am enthusiastic about this paper, which presents for the first time a protostomian PTH-like peptide with a functional analysis of both the peptide and its receptor genes. The manuscript is overall clearly written and the authors show convincing data which support the main conclusions of this paper.

I have some comments about the presentation of the results, but the authors have convinced me that they have discovered a bona fide insect PTH-like peptide and the functional analysis gives clear and interesting results. It is the first PTH of its kind in a protostomian species and as such makes it very interesting for the community of comparative neurobiologists and geneticists. The fact that PTH and its receptor seem to act on the regulation of insect exoskeleton formation, a function analogous to PTH and PTHLH function in bone homeostasis in mammals is appealing and may lay bases for deep homology conjectures, as it was done in other papers presenting functional analyses of conserved bilaterian neuropeptides including vasopressin (Beets et al 2012, PMID: 23112336) and TRH (Van Sinay et al. 2017, PMID: 28461507).

**Specific comments:**

On the introduction:

- lines 77-79 in the authors introduces the function of vertebrate PTH and its receptors but does not make any mention of the fact that there are three paralogous PTH-like peptide genes in human (PTH, PTHLH, TIP-39/PTH2), and as such are all three orthologous to iPTHs, according to the claims of this paper. While it has mostly been studied in fish and may be not essential here, it would be interesting to add one or a few lines on the different peptides.

- Then in lines 80-83 the authors write : “The PTH receptor lineage is traceable to before the time of the deuterostome-protostome split in the basal Bilateria, but there are no obvious homologous PTH ligands in the basal protostome lineages.” This is a statement which follows from the previous sentence on vertebrate PTH and PTHRs but without any reference supporting the ancestrality of PTHRs in bilaterian lineages, although there is a literature on the subject including Mirabeau and Joly 2013 (PMID:23671109), Jekely 2013 (PMID: 23637342), and Elphick, Mirabeau, Larhammar 2018 (PMID: 29440283). Also while it is correct that there are no known protostomian PTH-like peptides, these peptides are expected to occur in insects (as part of the larger glucagon+PTH-like group) as demonstrated in Mirabeau and Joly 2013 (PMID:23671109).

- Both Figure S1 and lines 125-126 (“The iPTHRs belong to the class B1 G protein-coupled receptors (GPCRs) and are clear orthologs of vertebrate PTHRs (Fig. 2A; Fig. S1).”) that are supported by this figure are problematic for the following reasons :

> the branch of interest here, leading to protostomian sequences and human PTHRs is not indicated as a branch supported (there is no circle on it), instead only the protostomian group of sequences is supported as a “monophyletic” group. It is probably a mislabeling of the branches or a misunderstanding from the authors or from me. Also the legend of figure S1 is succinct and does not point to a particular message.

> the method used here is the neighbour-joining method which is a fast and alignment-free method but less accurate than alignment and substitution model-based methods (Maximum likelihood-based for example), at least for this type of problems where robust and rather long alignments are possible to obtain. I would strongly recommend using this class of methods here. Also importantly it would be good to have a phylogram (which shows an estimation of the branch lengths) and not a cladogram (which shows only the topology of the tree). Like this we could assess for instance whether Tc-PTHR1 is more “basal” than Tc-PTHR2, as expected from the results of this paper.

> I understand that the main message here is to establish the orthology of vertebrate PTHRs to the protostomian sequences but there is a strong unbalance between the number and diversity of the two groups of sequences and the inclusion of additional vertebrate sequences could be used to improve the resolution of the tree. More importantly the outgroup is incomplete, the authors are right to choose protostomian sequences such as PDFR and DH-44Rs which are members of the secretin receptor family, but they leave out other members of the vertebrate secretin receptor family including secretin, PACAP, VIP, glucagon-like peptide receptors, which have been shown to be more closely related to vertebrate PTH receptors than the other outgroup sequences are (Mirabeau and Joly 2013, PMID:23671109) and that are better potential outgroup sequences. The authors may then find that it is difficult to establish a “one-to-one” orthology between vertebrate PTHRs and protostomian PTHRs and that the glucagon/PACAP/secretin/VIP receptor family must be included as paralogous sequences in the evolutionary model scenario.

- For the expression analysis it would appreciated to have either a table recapitulating the neurons at the different stages, with the fraction of individuals showing expression in the given regions, or even better a schematic drawing of the different stages with the individual neurons or regions of positive expression.

- The authors show strong phenotypes for the PTHRs mutants. The barplots in Fig. 6A-E show a more pronounced effect of Tc-PTHR1 inactivation, perhaps in line with the fact that iPTH binds and activates Tc-PTHR1 with a stronger affinity than it does with Tc-PTHR2. Although it seems to me that panel 6F shows on the contrary a stronger phenotype for PTHR2. Are the photos shown representative of the phenotypes recorded?

- lines 270-273: The sentence “However, the RNAi of the ligand Tc-iPTH did not show any phenotype. Q-PCR for examining the transcript level and immunohistochemistry indicated that the suppression of Tc-iPTH by the RNAi may have not been sufficient to induce a phenotype (Fig. 6A; Fig. S4 H to K).” implicitly suggests that Fig. 6A shows a less marked reduction of Tc-iPTH when the RNAi is applied, compared to the Tc-iPTHR1 and Tc-iPTHR2 knockdown experiments, and this does not appear to be the case (the inhibition seems to be of the same order of magnitude). This either may mean that the inhibition is not sufficient to cancel the effect of that gene while an inhibition of similar strength for the receptors has a stronger effect. This could be explained by the fact that peptides are produced in excess compared to their receptors or for some other reason, but this should be somehow discussed differently to account for the data presented. Another possibility could be that there is another PTH-like gene yet to be discovered in Tribolium that could bring some redundancy to the system? Also have the authors tried to design other miRNA to inhibit Tc-iPTH?

In addition negative controls for the immunohistochemistry are shown for Tc-iPTH and Tc-iPTHR1 (and the comparison seems to be in line with a more complete inhibition of Tc-iPTH than Tc-iPTHR1 as claimed by the authors – then maybe they should only cite that Figure and not Fig 6A) but no negative controls seem to be presented for Tc-iPTHR2.

Minor comment for Fig. 6: clarify what “Phenotype (%)” means in the Figure. I suggest being more specific: “Fraction (of animals) with phenotype (%)”. Also the legend of Fig. 6 does not specify whether numbers for “Adult morphological deficiency” are the percentage of animals with a deficiency among all the animals or only among those which went through ecdysis. I suggest using the latter definition as it may improve the visual resolution of the figure for that part of the data.

- lines 280-282: “Likewise, three dimensional scatter plots also showed differentially expressed genes in the dsiPTHR1 and dsiPTHR2 treatments showing similar patterns (Fig. 8B)”:

I find difficult to assess that with the 3D plot shown in Fig. 8B. I would suggest presenting 3 2D plots (Tc-iPTHR1 vs Tc-iPTHR2, Tc-iPTHR1 vs control, Tc-iPTHR2 vs control) and/or the number of genes DE in the 3 comparisons, to make it clear that the difference Tc-iPTHR1 vs Tc-iPTHR2 is small compared toTc-iPTHR1/2 vs control.

285-287: “Large portions of differentially expressed (DE) genes were common in response to the dsiPTHR1 and dsiPTHR2 treatments (110 and 242 genes for >4× up or down genes, respectively, Fig. 8D).” This is visible from the numbers of the Venn diagram, however it could be further quantified with statistics, for instance a Fisher test of independence.

- Paragraph of lines 303-310 and Figure 8D: Could the author give us a sense of the statistical significance of the results: for instance it seems that the term “extracellular structural components” is very much enriched, but it could be quantified in terms of actual enrichment, and/or p-value associated to an enrichment test - Fisher’s exact test for instance. Also the statistical significance of the overlap between PTHR1 and PTHR2-regulated genes, for both up- and down-regulation, could be quantified with a pvalue, although it is clear that the significance will be high.

- Paragraph of lines 321-238 and Figure 9: 4 chitinases are found up-regulated in the Tc-PTHRs mirna experiments, which would naively suggest a down-regulation of chitin content in these experiments, unlike what is presented in Fig. 9 (an up-regulation of chitin content). This discrepancy should perhaps be addressed in the discussion. More generally the possible/expected relationships between the expression of some of the cuticular genes found (like chitinases) and the phenotypes observed could be discussed more thoroughly to orient the reader.

Minor comments and corrections:

41: **sequence homologous to PTH** instead of **~~PTH homologous~~**

41: insect **genomes**, instead of insect **genome**

50: that **the** iPTH system

65: that **the** iPTH system

72: **the** development of new molecular techniques

96: “**with a lack of homology**” is awkward

114: “**Fig. 1C**” should read “**Fig 1B**”.

lines 126-128: The authors write “The evolutionary processes of vertebrate PTHRs have previously been described as the events of multiple gene duplications and deletions [4].”:

The reference here in a results section seems misplaced.

149-151: “Conservation of seven cysteines **(marked as stars on top of the alignment in S2A)** in the predicted extracellular loops including the N-terminal extracellular region implies a significant role of disulfide bridges for formation of the ligand binding pocket (Fig. S2).” I would add the following “**(marked as stars on top of the alignment in S2A)**” to better orient the reader.

178: “Expression patterns **in** different developmental stages.” Should read: “**at** different developmental stages”

231: “which did not ~~showing~~ **show** any staining in”

357: “development is enigmatic **yet**”. Should read: “is **still** enigmatic”

368: “three CPAP proteins here have been reported **to be** expressed only in cuticle-forming tissues”

371: **A similar** phenotype

372-373: **. Among** **down-regulated** extracellular **matrix**

376-377: In addition, **~~although~~**a number of chitinases were also up-regulated: Chitinase 8, 9, and 16, and 3-like, for which the RNAi phenotypes **~~are~~** **have** not yet **been** documented.

384: **~~metalolism~~** **metabolism**

387-338: the sentence “and culminated as the phenotype of defects in wing development in the adult eclosion [25].” needs to be rewritten. For instance: “and culminated **in animals with** a phenotype of defects in wing development in the adult eclosion [25].”

388-389: **ecdysone-inducible**

438: For **the** stage-specific expression study

443: For **the** tissue**-**specific expression study

444: total RNA... **was** also extracted.

467: “QPCR using a pool” (space missing)

510: replace sentence with: “GO **terms** for each T. castaneum genes **were** obtained”, or “**a GO term** for each T. castaneum genes **was** obtained”

511: replace “Only those with more than 4× differences were filtered” with something equivalent to “Only those with more than 4× differences were kept”

518: “**was collected**” instead of “**was collect**”

526: “**described**” instead of “**descried**”

Figure S1 “**Mollusa**” should read “**Mollusca**”

“Mammal” should read “**Mammalia**” for consistency

Figure S6: A: gene names risk not being readable with the current size. B Legends should be made bigger.

Line 585: **~~Triboliwn~~** **Tribolium** freemani, under crowded conditions. Insect Biochem Molec. 2018;101:76-84.

714-719: the legend names of the two tables should be “Dataset_S1” and “Dataset_S2”. In Dataset_S2 I do not relate the column names T3a-RPKM and T2b-RPKM to measurements that were done. I assume that they correspond to dsiPTHR1-RPKM and dsiPTHR2-RPKM as in Dataset_S1, if so it would be useful to make the corresponding modification.
