# Supplementary material for: A new neuropeptide insect parathyroid hormone iPTH in the red flour beetle Tribolium castaneum
Source: PLoS Genet. 2020 May 4;16(5):e1008772. doi: 10.1371/journal.pgen.1008772 (PMC7224569; doi:10.1371/journal.pgen.1008772)
Supplement: Attachment — Submitted filename: Response to Reviewers.docx [file pgen.1008772.s015.docx]

Dear Dr. Schoofs, Dr. Barsh and Reviewers,

We are greatly thankful for your attention and useful comments and suggestions on our manuscript entitled “A new neuropeptide insect parathyroid hormone iPTH in the red flour beetle *Tribolium castaneum*” (PGENETICS-D-19-01925).

The comments are very valuable and helpful for revising and improving our paper. We have studied the comments carefully and have revised the manuscript according to the detailed suggestions. Clean copy and marked-up copy of the manuscript files have been resubmitted to your journal, named “Manuscript” and “Related Manuscript”, respectively. The main corrections in the manuscript and the responses to the reviewers’ comments are listed below point by point (Note information is based on the line numbers in “Related Manuscript” file).

We hope it could be a satisfactory revision and welcome any other comments from you. If you have any question about this paper, please don’t hesitate to let me know.

Thank you again and best regards!

Sincerely yours,

Bin Li

Detailed response to reviewer’s comments:

**Reviewer #1: Comments:**

Introduction
line [87] Add other references of relevant insect GPCR papers/reviews?

**Response:** Line 97-98, we added other two references here [1, 2]:

Harmar AJ. Family-B G-protein-coupled receptors. Genome Biol. 2001;2(12):REVIEWS3013.

Tanaka Y, Suetsugu Y, Yamamoto K, Noda H, Shinoda T. Transcriptome analysis of neuropeptides and G-protein coupled receptors (GPCRs) for neuropeptides in the brown planthopper Nilaparvata lugens. Peptides. 2014;53:125-33.

Results
[129] Change (Fig. 2A) to (Fig. 2A; Fig. S1). Figure S1 displays the gene duplication in insects more clearly.

**Response:** Line 143-144, we have changed it to (Fig. 2A; Fig. S1). In Addition, the duplication in Fig.S1 is marked as red in the current version.

[136] According to the supporting information file, Fig S1 only displays PTHRs and no iPTH sequences. Thus, there is either a reference to a Figure missing or a reference to literature.

**Response:** Line 150, citation of Table S1 is added (Fig. S1; Table S1), where the Table S1 is the PTH sequences for different species fetched from the NCBI database.

[149-151] This is described in literature as characteristic of B-group GPCRs. Add this fact in your description/discussion, together with relevant references.

**Response:** Line 166-167, a sentence was added as,

“Stabilization of extracellular structure by disulfide bonds in GPCR-B has previously shown [3-6].”

[166-167] Remove line (see the definition of early and late in the materials and methods), as it speaks for itself that the description would and should be in this section.

**Response:** Line 182-183, the line is now removed. ~~“(see the definition of early and late in the materials and methods)~~”

Discussion
[346-349] Agreed, this is a very interesting finding. However, how does this correlate with the neural projections of iPTH (Fig. 4) and the expression levels of both iPTH and both iPTHR (as described in Fig. 3 & 5)? Both receptors and iPTH itself seem to be mainly expressed in Gut and CNS (Fig 3.). There should be at least mention of this fact in your discussion, even if the effect would probably be indirect.

**Response:** We agree with this reviewer. Line 470-475, we modified the conclusion, second sentence from the last of the Discussion, to incorporate the question remaining as (underlines are for addition):

The functions of this peptidergic system investigated by RNAi include the role as a master regulator of cuticle maturation. High expressions of the iPTH and its receptors in the gut and the nervous system indicate that additional functions are likely. Molecular and cellular evaluation of the further direct and indirect functions of iPTH awaits further studies.

[354-355] You have not really proven that the receptors are unable to compensate each other, see comment line 460-462. Suggested experiment: RT-qPCR of both receptors in experimental tissues used of these experiments. As you already have the samples, this addition should be easily conducted. These results must be added in supplementary data. Or alternatively, show this fact explicitly in a figure separate from of your RNAseq data.

**Response:** The RNAi efficiency data in both larval and pupal stage could prove that the receptors are unable to compensate each other: Fig.6A showed that the dsRNA injection in the larval stage for each receptor specifically suppressed the target gene and did not cause any expression changes of the other receptor. In addition, we also have a similar pattern after pupal injection as the larval RNAi (Figure below), which is not included in the manuscript due to the limited space.

Materials and methods
[439] Define “multiple individuals” as this could describe any quantity, from 2 to infinity. The reader should be informed about the size of the biological pool by which one can determine the relevance of the described results.

**Response:** Lines 498-502, the specific quantities of the “multiple individuals” are marked in the brackets of the following sentences:

“early embryo (EE, 1 day old, ~50 mg), late embryo (LE, 3 day old, ~50 mg), early larva (EL, 1 day old, ~50mg), late larva (LL, ~15 days old, 3 individuals), early pupa (EP, 1 day old, 3 individuals), late pupa (LP, 5 days old, 3 individuals), early adult (EA, 1 day old, 3 individuals), and late adult (LA, 10 days old, 3 individuals)”.

[460-462] This potential has not been experimentally excluded, or it is not shown. One should. What kind of controls or additional experiments have been performed to confirm the data and to show that these are not due to off-target effects?

**Response:** The main two reasons of off-target effects are the non-specific sequence of the dsRNA and the regulation of endogenous RNA caused by exogenous dsRNA. For the first aspect, we have investigated the specificity of dsRNA region using blast in *T. castaneum* genome. Except the gene itself, the best matched sequence with the dsRNA of *iPTHR1* and *iPTHR2* is a section of 18 and 17 consecutive nucleotide sequences, respectively. These best matches are shorter than the length of the small fragments of dsRNA (21-23bp), and did not reach the length (≥19-nucleotide perfect matches) that may contribute to off-target effects[7]. Most of all, our RT-PCR after RNAi supports that the suppression was sub-type specific without cross reactivity to each other as was shown in answers to the above comment of the reviewer.

For the second aspect, typically, the green fluorescent protein (GFP) dsRNA or other dsRNA such as ds-vermillion could serve as a positive control in RNAi analysis. In previous studies, injection of ds-GFP into *T. castaneum* did not result in any phenotype, and injection ds-vermillion into *T. castaneum* just changed the eye color without affecting the development of the beetles [8, 9]. In our laboratory, we also did this experiment and confirmed that injection of ds-GFP and ds-vermillion into the *T. castaneum* 15 days’ larvae in an equal volume did not influence the development of beetles, which is the same as injection of IB (buffer). This implied that the results we observed in *iPTHR* RNAi analysis were all due to suppression of the specific target genes expression, not because of the presence of dsRNA.

However, we did find that the ds-vermillion injection produces a heat resistance phenotype[10]. In addition, ds-GFP injections in multiple experiments resulted somewhat irregular data for egg numbers although it never showed the abnormal phenotypes in the development. Conclusively, we performed the control experiments with buffer injections, but not with dsRNAs which are potentially producing unexplained, unmeasured and unpredictable effects. Avoiding the unknown effects in the control was more important in the NGS for DE analyses. Therefore, the current study used consistently buffer injection as the control. Indeed, in addition to the buffer injection controls, which serve as the injection would and osmotic shock control, our treatments, such as dsRNA for PTH, function as a good negative control without the phenotype that we focused.

An important additional experiment that we performed to address this question was two dsRNAs tested for each gene. We have designed two dsRNAs in different region to test the RNAi phenotypes. The different regions of ds*iPTHR1* are: positions 76 to 492 and 655 to 1259 in the CDS, and different regions of ds*iPTHR2* are: positions 238 to 673 and 1198 to 1629 in the CDS, respectively. Resulting phenotypes and specificities of the RNAi were similar as were described in the manuscript supporting the results in the manuscript although the percent individuals affected varied in the experiments.

Ds-GFP	ds-vermillion

[466-467] This RNAi efficiency at RNA/protein level is not shown. Add to Supplementary data.

**Response:** We showed RNAi efficiency data at RNA level in Fig.6A. It proved that RNAi of *Tc-iPTHR1* and *Tc-iPTHR2* has successfully suppressed the expression of their specific target gene.

**Reviewer #2**

**General comments:**

I am enthusiastic about this paper, which presents for the first time a protostomian PTH-like peptide with a functional analysis of both the peptide and its receptor genes. The manuscript is overall clearly written and the authors show convincing data which support the main conclusions of this paper.

I have some comments about the presentation of the results, but the authors have convinced me that they have discovered a bona fide insect PTH-like peptide and the functional analysis gives clear and interesting results. It is the first PTH of its kind in a protostomian species and as such makes it very interesting for the community of comparative neurobiologists and geneticists. The fact that PTH and its receptor seem to act on the regulation of insect exoskeleton formation, a function analogous to PTH and PTHLH function in bone homeostasis in mammals is appealing and may lay bases for deep homology conjectures, as it was done in other papers presenting functional analyses of conserved bilaterian neuropeptides including vasopressin (Beets et al 2012, PMID: 23112336) and TRH (Van Sinay et al. 2017, PMID: 28461507).

**Response:** We appreciate the reviewer’s incisive comments. Now, in Line 83-84, we added a sentence in the introduction as,

“Functional studies of ancestral bilaterian neuropeptides have been successful by starting from the sequence similarities, e.g., vasopressin [11-14].”

**Specific comments:**

On the introduction:

- lines 77-79 in the authors introduces the function of vertebrate PTH and its receptors but does not make any mention of the fact that there are three paralogous PTH-like peptide genes in human (PTH, PTHLH, TIP-39/PTH2), and as such are all three orthologous to iPTHs, according to the claims of this paper. While it has mostly been studied in fish and may be not essential here, it would be interesting to add one or a few lines on the different peptides.

**Response:** Line 89, a line is added to describe the evolution of the ligand PTH as,

“Likewise, the ligand PTH also has undergone gene duplication and losses in vertebrates [15].”

- Then in lines 80-83 the authors write: “The PTH receptor lineage is traceable to before the time of the deuterostome-protostome split in the basal Bilateria, but there are no obvious homologous PTH ligands in the basal protostome lineages.” This is a statement which follows from the previous sentence on vertebrate PTH and PTHRs but without any reference supporting the ancestrality of PTHRs in bilaterian lineages, although there is a literature on the subject including Mirabeau and Joly 2013 (PMID:23671109), Jekely 2013 (PMID: 23637342), and Elphick, Mirabeau, Larhammar 2018 (PMID: 29440283). Also while it is correct that there are no known protostomian PTH-like peptides, these peptides are expected to occur in insects (as part of the larger glucagon+PTH-like group) as demonstrated in Mirabeau and Joly 2013 (PMID:23671109).

**Response:** Line 90-91, we added the literatures on the existing sentences, mentioning the ancestry of PTH receptor as,

“The PTH receptor lineage is traceable to before the time of the deuterostome-protostome split in the basal Bilateria [16-18], but. . . .”

- Both Figure S1 and lines 125-126 (“The iPTHRs belong to the class B1 G protein-coupled receptors (GPCRs) and are clear orthologs of vertebrate PTHRs (Fig. 2A; Fig. S1).”) that are supported by this figure are problematic for the following reasons :

> the branch of interest here, leading to protostomian sequences and human PTHRs is not indicated as a branch supported (there is no circle on it), instead only the protostomian group of sequences is supported as a “monophyletic” group. It is probably a mislabeling of the branches or a misunderstanding from the authors or from me. Also the legend of figure S1 is succinct and does not point to a particular message.

> the method used here is the neighbour-joining method which is a fast and alignment-free method but less accurate than alignment and substitution model-based methods (Maximum likelihood-based for example), at least for this type of problems where robust and rather long alignments are possible to obtain. I would strongly recommend using this class of methods here. Also importantly it would be good to have a phylogram (which shows an estimation of the branch lengths) and not a cladogram (which shows only the topology of the tree). Like this we could assess for instance whether Tc-PTHR1 is more “basal” than Tc-PTHR2, as expected from the results of this paper.

> I understand that the main message here is to establish the orthology of vertebrate PTHRs to the protostomian sequences but there is a strong unbalance between the number and diversity of the two groups of sequences and the inclusion of additional vertebrate sequences could be used to improve the resolution of the tree. More importantly the outgroup is incomplete, the authors are right to choose protostomian sequences such as PDFR and DH-44Rs which are members of the secretin receptor family, but they leave out other members of the vertebrate secretin receptor family including secretin, PACAP, VIP, glucagon-like peptide receptors, which have been shown to be more closely related to vertebrate PTH receptors than the other outgroup sequences are (Mirabeau and Joly 2013, PMID:23671109) and that are better potential outgroup sequences. The authors may then find that it is difficult to establish a “one-to-one” orthology between vertebrate PTHRs and protostomian PTHRs and that the glucagon/PACAP/secretin/VIP receptor family must be included as paralogous sequences in the evolutionary model scenario.

**Response:** We appreciate the reviewer’s comments.

First, we added more vertebrate sequences of PTHRs to construct the phylogenetic tree. Second, vertebrate secretin receptor family including secretin, PACAP, VIP, glucagon-like peptide receptors also has been added. As shown in the modified Fig. S1, the reviewer's prediction is completely correct, we indeed could not establish a “one-to-one” orthology between vertebrate PTHRs and protostomian PTHRs and the glucagon/PACAP/secretin/VIP receptor family. Thus, we changed our description as below: “The iPTHRs belong to the class B1 G protein-coupled receptors (GPCRs) and are considered counterparts of vertebrate PTHRs based on sequence similarity”.

- For the expression analysis it would appreciated to have either a table recapitulating the neurons at the different stages, with the fraction of individuals showing expression in the given regions, or even better a schematic drawing of the different stages with the individual neurons or regions of positive expression.

**Response:** Because the expression pattern of iPTH in both CNS and gut was similar in different stages, we here added a schematic drawing (Fig. 4 R-U) to show the positive expression regions of iPTH in the different parts of CNS and midgut. We also added additional description on the result of immunohistochemistry by adding words as:

Line220-223, “Diagrams of the brain (R), ganglion (S) showed the neuronal cells and axonal projections in CNS. Diagrams of anterior part of midgut (T) showed stomatogastric nervous projections. The posterior part of midgut (U) showed the enteroendocrine cells.”

Line227-228, “which are similar to the bipolar enteric plexus cells in *Manduca sexta,*[19].”

- The authors show strong phenotypes for the PTHRs mutants. The barplots in Fig. 6A-E show a more pronounced effect of Tc-PTHR1 inactivation, perhaps in line with the fact that iPTH binds and activates Tc-PTHR1 with a stronger affinity than it does with Tc-PTHR2. Although it seems to me that panel 6F shows on the contrary a stronger phenotype for PTHR2. Are the photos shown representative of the phenotypes recorded?

**Response:** In fact, there are variations among individual phenotypes, while in general, the photos shown here is representative of the phenotypes. The phenotype of ds*iPTHR1* in Fig. 6F represents 81.3±8.4% of individuals getting ecdysial arrest at the time of adult eclosion, while the phenotype of ds*iPTHR2* in Fig. 6F represents 43.8±10.5% individuals with the phenotype arrested at the time of eclosion after larval injection. And it also can represent 28.6±5.5%, 5.4±0.2% individuals with the same phenotype after pupal injection of ds*iPTHR1* and ds*iPTHR2*, respectively.

In order to improve the clarify, we added the phenotype description “ecdysial arrest” on the top of Fig. 6F, and the percentage of this phenotype is described in Fig. 6B and Fig. 6C.

- lines 270-273: The sentence “However, the RNAi of the ligand Tc-iPTH did not show any phenotype. Q-PCR for examining the transcript level and immunohistochemistry indicated that the suppression of Tc-iPTH by the RNAi may have not been sufficient to induce a phenotype (Fig. 6A; Fig. S4 H to K).” implicitly suggests that Fig. 6A shows a less marked reduction of Tc-iPTH when the RNAi is applied, compared to the Tc-iPTHR1 and Tc-iPTHR2 knockdown experiments, and this does not appear to be the case (the inhibition seems to be of the same order of magnitude). This either may mean that the inhibition is not sufficient to cancel the effect of that gene while an inhibition of similar strength for the receptors has a stronger effect. This could be explained by the fact that peptides are produced in excess compared to their receptors or for some other reason, but this should be somehow discussed differently to account for the data presented. Another possibility could be that there is another PTH-like gene yet to be discovered in Tribolium that could bring some redundancy to the system? Also have the authors tried to design other miRNA to inhibit Tc-iPTH?

In addition negative controls for the immunohistochemistry are shown for Tc-iPTH and Tc-iPTHR1 (and the comparison seems to be in line with a more complete inhibition of Tc-iPTH than Tc-iPTHR1 as claimed by the authors – then maybe they should only cite that Figure and not Fig 6A) but no negative controls seem to be presented for Tc-iPTHR2.

**Response:** We appreciate the reviewer’s helpful comments. We have added the explanations the reviewer pointed to the discussion (Lines 457-459; Lines 463-465).

Line 457-459, “although Q-PCR result showed a similar marked reduction of both Tc-iPTH and its receptors when the RNAi is applied, the inhibition may have not been sufficient for the phenotype in the RNAi of Tc-iPTH as the dsRNA of Tc-iPTH did not show any phenotype.”

Line 463-465, “An alternative possibility is that there may be another ligand for PTH receptors that yet needs to be discovered in *T. castaneum*.”

Our original intention was to say that both the level of Q-PCR and immunohistochemistry indicate that there is still some remaining of iPTH, but we agree with your suggestion that compare the Q-PCR result and phenotype of the ligand and receptors. However, it is hard to draw the conclusion that “Q-PCR for examining the transcript level indicated that the suppression of Tc-iPTH may have not been sufficient to induce a phenotype” from the Q-PCR data. Thus, we changed the sentence to “~~Q-PCR for examining the transcript level and~~ Immunohistochemistry indicated that the suppression of Tc-iPTH by the RNAi may have not been sufficient to induce a phenotype (~~Fig. 6A;~~ Fig. S4 H to K) ”.

We haven’t try miRNA to inhibit the Tc-iPTH, the reasons are as below: First, *T. castaneum* is well known in showing a strong systemic RNAi effect to the injection of dsRNA. Second, dsRNA is more effective at producing RNA interference (RNAi) than sense or antisense RNA. Third, we have performed *Tc-iPTH* RNAi by using three different partially overlapping regions of dsRNA, and the longest dsRNA contains the whole CDS region and the 5’UTR and 3’UTR region, which is quite longer than miRNA. In this case, there is still no significant phenotype in the ds*iPTH* group.

We also have tried to detect the distribution of iPTHR2. However, our antibody of R2 did not work, so we focused on the receptor iPTHR1 which showed strong reactivity with iPTH.

Minor comment for Fig. 6: clarify what “Phenotype (%)” means in the Figure. I suggest being more specific: “Fraction (of animals) with phenotype (%)”. Also the legend of Fig. 6 does not specify whether numbers for “Adult morphological deficiency” are the percentage of animals with a deficiency among all the animals or only among those which went through ecdysis. I suggest using the latter definition as it may improve the visual resolution of the figure for that part of the data.

**Response:** We changed “Phenotype (%)” to “Fraction with phenotype (%)” in Fig. 6B and Fig. 6C.

The percentage of “Adult morphological deficiency” in Fig. 6B and C are the percentage of animals with a deficiency among all the animals. In order to improve the clarity of the figure, we changed the original Fig. 6B and C to a “stack column” form and added a further description in the text and legend. Also, we added the corresponding notes on the top of the Fig. 6F and Fig. 7A and 7B.

- lines 280-282: “Likewise, three dimensional scatter plots also showed differentially expressed genes in the dsiPTHR1 and dsiPTHR2 treatments showing similar patterns (Fig. 8B)”:

I find difficult to assess that with the 3D plot shown in Fig. 8B. I would suggest presenting 3 2D plots (Tc-iPTHR1 vs Tc-iPTHR2, Tc-iPTHR1 vs control, Tc-iPTHR2 vs control) and/or the number of genes DE in the 3 comparisons, to make it clear that the difference Tc-iPTHR1 vs Tc-iPTHR2 is small compared toTc-iPTHR1/2 vs control.

**Response:** We agree that the 3D plot is difficult to visualize. We added the 2D plots in the Supplementary data (Fig. S6) while the 3D plot is left as it was because the 3D plot is the best way to show that the DE genes are common in both RNAi of *Tc-iPTHR1* and *Tc-iPTHR2*.

285-287: “Large portions of differentially expressed (DE) genes were common in response to the dsiPTHR1 and dsiPTHR2 treatments (110 and 242 genes for >4× up or down genes, respectively, Fig. 8D).” This is visible from the numbers of the Venn diagram, however it could be further quantified with statistics, for instance a Fisher test of independence.

**Response:** We added the R-square for the correlation between the DE shown in the plot Fig. 8C in the linear regression showing 0.78, high correlatedness. The text (Line 310-311) and figure (Fig. 8C) are modified for inserting R-square value in Fig. 8C.

- Paragraph of lines 303-310 and Figure 8D: Could the author give us a sense of the statistical significance of the results: for instance it seems that the term “extracellular structural components” is very much enriched, but it could be quantified in terms of actual enrichment, and/or p-value associated to an enrichment test - Fisher’s exact test for instance. Also the statistical significance of the overlap between PTHR1 and PTHR2-regulated genes, for both up- and down-regulation, could be quantified with a pvalue, although it is clear that the significance will be high.

**Response:** The categories of gene functions were manually made in addition to the gene functions assigned by the Blast2Go program. This was due to incomplete GO term assignments and errors included in the B2G program that was manually corrected. For example, the “extracellular structural components” includes structural components of cuticle and other extracellular matrix proteins manually selected for a better presentation. Although we believe that the outcome has significance in the statistics, we were unable to numerize the significance value because we do not know the error rate in the GO term assignment in the entire gene predictions while we made manual corrections only for the DE genes.

- Paragraph of lines 321-238 and Figure 9: 4 chitinases are found up-regulated in the Tc-PTHRs mirna experiments, which would naively suggest a down-regulation of chitin content in these experiments, unlike what is presented in Fig. 9 (an up-regulation of chitin content). This discrepancy should perhaps be addressed in the discussion. More generally the possible/expected relationships between the expression of some of the cuticular genes found (like chitinases) and the phenotypes observed could be discussed more thoroughly to orient the reader.

**Response:** In our RNA-seq data, we found the down-regulated *chitinase 5* and *chitinase 10* which were previously shown to be affecting degradation of old cuticle at the molting stage, ultimately leading to molting deficiency in *T. castaneum* (Line 392-393) [20, 21]. Although there are four up-regulated chitinases captured in the RNA-seq data, chitinase 8 contains only one chitin binding domain while Chitinase 9 and 16 lacked this domain. In addition, knockdown these three chitinases have no significant abnormalities in *T. castaneum*, which may suggest that they are not essential for *T. castaneum* development and may have no effect on the cuticle formation, while the phenotypes of overexpressions of these genes are potentially disruptive in *T. castaneum* development.

We added the relative discussion of the up-regulated chitinases in the text in Line 422-428 as below:

“In addition, up-regulated expressions of *Chitinase 8*, *9*, *16*, and *3-like* were found after RNAi of *Tc-iPTHRs*, however, three of them (*Chitinase 8*, *9*, and *16*) belong to the Group IV chitinases, in which *Chitinase 8* contains only one chitin binding domain while *Chitinase 9* and *16* lacked this domain [22]. It also had been reported there were no detectable abnormalities after RNAi of these three chitinases [20]. These results suggested that they are not essential while the phenotypes of overexpressions of these genes are potentially disruptive in *T. castaneum* development.”

Minor comments and corrections:

41: **sequence homologous to PTH** instead of **~~PTH homologous~~**

**Response:** Line 41: **~~PTH homologous~~** has been changed to “**sequence homologous to PTH**”

41: insect **genomes**, instead of insect **genome**

**Response:** Line 42: “insect **genome**” has been changed to “insect **genomes**”

50: that **the** iPTH system

**Response: “the”** has been added before “iPTH system” in Line 51, 72, and other places in the whole text.

65: that **the** iPTH system

**Response: “the”** has been added before “iPTH system” in Line 51, 72, and other places in the whole text.

72: **the** development of new molecular techniques

**Response:** Line 78: **“the”** has been added before “development of new molecular techniques”

96: “**with a lack of homology**” is awkward

**Response:** Line 106: “with a lack of homology to” has been changed to “**with the lack of**”

114: “**Fig. 1C**” should read “**Fig 1B**”.

**Response:** Line 126: **“Fig. 1C”** has been changed to “**Fig 1B**”

lines 126-128: The authors write “The evolutionary processes of vertebrate PTHRs have previously been described as the events of multiple gene duplications and deletions [4].”:

The reference here in a results section seems misplaced.

**Response:** Line 142: We changed the references [23, 24].

149-151: “Conservation of seven cysteines **(marked as stars on top of the alignment in S2A)** in the predicted extracellular loops including the N-terminal extracellular region implies a significant role of disulfide bridges for formation of the ligand binding pocket (Fig. S2).” I would add the following “**(marked as stars on top of the alignment in S2A)**” to better orient the reader.

**Response:** Line 164: “**(marked as stars on top of the alignment in Fig. S2A)**” has been added to better orient the reader.

178: “Expression patterns **in** different developmental stages.” Should read: “**at** different developmental stages”

**Response:** Line 194: “**in** different developmental stages” has been changed to “**at** different developmental stages”

231: “which did not ~~showing~~ **show** any staining in”

**Response:** Line 252: “which did not **showing**” has been changed to “which did not **show**”.

357: “development is enigmatic **yet**”. Should read: “is **still** enigmatic”

**Response:** Line 386: “is enigmatic **yet**” has been changed to “is **still** enigmatic”

368: “three CPAP proteins here have been reported **to be** expressed only in cuticle-forming tissues”

**Response:** Line 406: This sentence has been revised into “three CPAP proteins here have been reported **to be** expressed…”

371: **A similar** phenotype

**Response:** Line 410-411: “Similar phenotype” has been changed to “A similar phenotype”.

372-373: **. Among** **down-regulated** extracellular **matrix**

**Response:** Line 411-412: “**down regulated**” has been changed to “**down-regulated**” here and in other places of the whole text and datasets. “**matix**” has been corrected to “**matrix**”.

376-377: In addition, **~~although~~**a number of chitinases were also up-regulated: Chitinase 8, 9, and 16, and 3-like, for which the RNAi phenotypes **~~are~~** **have** not yet **been** documented.

**Response:** Line 422-426: In order to discuss the results about some up-regulated chitinases after *iPTHRs* RNAi, this sentence has been revised to “In addition, up-regulated expressions of *Chitinase 8*, *9*, *16*, and *3-like* were found after RNAi of *Tc-iPTHRs*, however, three of them (*Chitinase 8*, *9*, and *16*) belong to the Group IV chitinases, in which *Chitinase 8* contains only one chitin binding domain while *Chitinase 9* and *16* lacked this domain [22]. It was reported there were no detectable abnormalities after RNAi of these three chitinases individually [20].”

384: **~~metalolism~~** **metabolism**

**Response:** Line 435: “**metalolism**” has been corrected to “**metabolism**”.

387-338: the sentence “and culminated as the phenotype of defects in wing development in the adult eclosion [25].” needs to be rewritten. For instance: “and culminated **in animals with** a phenotype of defects in wing development in the adult eclosion [25].”

**Response:** Line 439-440: This sentence has been rewritten as “and its knockdown culminated **in insects with a** phenotype of defects in wing development in the adult eclosion”

388-389: **ecdysone-inducible**

**Response:** Line 440-441: “**ecdysone inducible**” has been revised as “**ecdysone-inducible**”

438: For **the** stage-specific expression study

**Response:** Line 497: “**the**” has been added before “stage-specific expression study”

443: For **the** tissue**-**specific expression study

**Response:** Line 502: “**the**”and “**-**” has been added.

444: total RNA... **was** also extracted.

**Response:** Line 503: “**were**” has been changed to “**was**”

467: “QPCR using a pool” (space missing)

**Response:** Line 526: Space was added here.

510: replace sentence with: “GO **terms** for each T. castaneum genes **were** obtained”, or “**a GO term** for each T. castaneum genes **was** obtained”

**Response:** Line 569: We have revised the sentence to “GO **terms** for each T. castaneum genes **were** obtained”.

511: replace “Only those with more than 4× differences were filtered” with something equivalent to “Only those with more than 4× differences were kept”

**Response:** Line 570-571: The sentence has been replaced with “Only those with more than 4× differences were **kept**”.

518: “**was collected**” instead of “**was collect**”

**Response:** Line 577: We have corrected it to “**was collected**”.

526: “**described**” instead of “**descried**”

**Response:** Line 586: “**descried**” has been changed to “**described**”

Figure S1 “**Mollusa**” should read “**Mollusca**”

“Mammal” should read “**Mammalia**” for consistency

**Response:** In new Fig. S1, “**Mollusa**” has been changed to “**Mollusca**”; “Mammal” has been changed to “**Mammalia**”；“**blattodia**”has been changed to “**blattodea**”

Figure S6: A: gene names risk not being readable with the current size. B Legends should be made bigger.

**Response:** We divided Fig. S6 into two figures (Fig. S7 and Fig. S8). In this case, the gene names in new Fig. S7 are bigger to be readable. The legends in new Fig. S8 have been changed to a bigger size.

Line 585: **~~Triboliwn~~** **Tribolium** freemani, under crowded conditions. Insect Biochem Molec. 2018; 101: 76-84.

**Response:** Line 682: This reference has been corrected.

714-719: the legend names of the two tables should be “Dataset_S1” and “Dataset_S2”. In Dataset_S2 I do not relate the column names T3a-RPKM and T2b-RPKM to measurements that were done. I assume that they correspond to dsiPTHR1-RPKM and dsiPTHR2-RPKM as in Dataset_S1, if so it would be useful to make the corresponding modification.

**Response:** Thanks for your careful review with the dataset. Yes, it should be “ds*iPTHR1*-RPKM” and “ds*iPTHR2*-RPKM”, and we have corrected it.

**Reviewer #3:**

This paper describes the first identification of insect peptides that activate “orphan” receptors (iPTHRs) homologous to the vertebrate parathyroid hormone receptor (PTHR). On this basis, the authors designate these peptides “insect parathyroid hormones” (iPTHs). Interestingly, while vertebrate PTH regulates calcium homeostasis by modulating bone metabolism, iPTHs are implicated in regulation of exoskeleton maturation and fecundity. This is a major advance in assigning functional roles for iPTHRs and likely will further our understanding of ligand-receptor coevolution. The manuscript summarizes a great deal of work and is well presented. The bioinformatic analysis is excellent and RNAi knockdown creates phenotypes that involve integumentary deficits. Some questions remain should be addressed in the interests of clarifying the source(s) and targets of iPTHs.

Specific Comments
1. Designation of peptides as iPTHs. On one hand, it is understandable, since functions of iPTHs and vertebrate PTHs appear to be quite different. But why not simply refer to them according to species (e.g., TcPTH)? Perhaps the authors could explain the rationale for the designation of iPTH.

**Response:** Our intention of using the word iPTH is for generalization of the group of peptides that were newly identified in this study. These peptides are not similar to the vertebrate PTH, even unsure for the evolutionary relatedness. Therefore, we used the word iPTH for the insect and invertebrate PTH to distinguish them from the PTH. In the meanwhile, we used the word Tc-iPTH in appropriate placed where the specific experiments are emphasized to avoid the confusions caused by the naming.

2. Sequences shown in Fig. E 1 fail to depict amidation at the C-terminus; it might be an idea to add a lower case “a” for amidation at the C-terminus of each sequence.

**Response:** In Fig. 1B, we have added a lower case “a” for amidation at the C-terminus of each sequence.

3. No mention of sequence similarity between iPTHs and vertebrate PTH. This reviewer found 47% similarity and 31% identity in a pairwise comparison with the consensus sequence shown in Fig. 1C. Addition of such information would complement analysis of sequence similarities of iPTHR and vertebrate PTHR.

**Response:** Thanks for the suggestion. Fig. 1C showed the sequence logo of conserved residues in the predicted mature peptides in various insect species, which indicated a high to moderate levels identity in the insects group. Upon your suggestion, we attempted to align and compare Tc-iPTH and the ligands in human. It showed that iPTH of *T. castaneum* just had an identity of 10.5%, 7.7%, and 11.9% with PTH, PTHrP (PTH related peptide) and TIP39 in *Homo sapiens*, respectively. Moreover, the identity between Tc-iPTHRs and PTHRs in *Homo sapiens* has been reported by our lab before, thus, we did not mention it here. In that paper, it is reported that Tc-PTHR1 shares 31%, 37% sequence identity with PTHR1 (NP_000307) and 35%, 36% identity with PTHR2 (NP_005039) of *Homo sapiens*, respectively. These data together suggest that the ligand has a larger divergence between invertebrate and vertebrate than the receptors. The corresponding comparison between Tc-iPTH and ligands in *Homo sapiens* has been added in the text: Line 122-123, “Although this mature peptide has no sequence homology to vertebrate PTH (lower than 12% identities with PTH, PTHrP, and Tip39 of *Homo sapiens*), highly conserved iPTHs were found in various insects and other taxa”.

4. Is there any evidence that iPTHs regulate hemolymph Ca levels, as is the case for vertebrate PTH?

**Response:** This is an interesting idea. However, this is beyond of our scope in this set of experiment. We could not make logical link of calcium levels to cuticle phenotype at this time while it is added on our to do list in the future.

5. Unlike the tissue source of vertebrate PTH, which is a well-defined gland behind the thyroid gland, iPTHs apparently originate from diverse sources (CNS, gut, etc). This is a matter of some uncertainty, however, since the antiserum used to locate iPTH-positive cells is not well characterized (see below). Hence, the possibility of cross-reactivity with other epitopes cannot be ruled out. Control experiments reported here are confined to negative results with pre-immune serum. This is not convincing. Additional controls could be: 1) determine if staining intensity decreases following RNAi of ligands and receptors. Although this has been shown to some extent in Fig. S4 for iPTH, convincing side-by-side comparisons with and without dsRNA are not provided. Another control could be pre-absorption of antiserum with ligand, following by staining, which should be negative. Likewise, no characterization of antisera raised against iPTHR is provided. Difficulties in specific immunohistochemical identification of GPCRs are well-known. Finally, it would be much more convincing if corroborating in situ hybridizations were included to demonstrate presence of the predicted transcripts for iPTHs and iPTHRs.

**Response:** We admit that immunohistochemistry of *T. castaneum* was highly challenging and the results we presented are summary/representation of multiple trials including the samples with ambiguous results. As we discussed in the manuscript, the consistent difficulties for obtaining nice immunohistochemistry in neuropeptide in this species was the main reason. In the revision, we added the reduced immunoreactivities, but remaining reactivities in the samples with *iPTH* RNAi in the supplementary data (Fig. S4 L-O). However, we were unable to quantify the results due to the variations among samples and high noise levels in certain trials. Therefore, we provide the representative pictures lacking the numerical comparisons.

6. A second problem with the immunohistochemical data shown in Figs 4 and 5 is that the images are not described in enough detail. It is often difficult to discern which parts of the nervous system are depicted in Figs. They are simply referred to en masse; consequently, the reader cannot discern anything specific.

**Response:** We have made a diagram to show the detail patterns of the immunohistochemical data in Fig. 4. We also added explanation to enforce the descriptions of immunoreactivities of Fig. 4 and Fig. 5 as,

Line220-223 “Diagrams of the brain (R), ganglion (S) showed the neuronal cells and axonal projections in CNS. Diagrams of anterior part of midgut (T) showed stomatogastric nervous projections. The posterior part of midgut (U) showed the enteroendocrine cells.”

Line227-228, “which are similar to the bipolar enteric plexus cells in *Manduca sexta*,[19].”

Line 246-247, “The immunoreactivity in the gut showed large stomatogastric neural cell bodies located on the anterior tip of midgut”

7. Any indication that iPTH is secreted by endocrine cells or glands, as is vertebrate PTH?

**Response:** Our immunohistochemistry included major insect endocine tissues, presumed corpora cardiaca and corpora allata, which were negative in the immunohistochemistry. We were able to detect the enteroendocrine cells, as described in the manuscript. However, secretion of the iPTH from the enteroendocrine cells will require a careful set of experiment including development of hemolymph ELISA. We were unable to move on this direction yet in this manuscript.

8. With regard to functionality of iPTH signaling, no mention is made in the abstract about reduction of fecundity following RNAi treatment.

**Response:** We added this part in the abstract, author summary and legend of Fig. 6.

Abstract (Line 53-54): “Moreover, RNAi of *iPTHRs* also led defects in fecundity with significant reductions in egg numbers and hatching rates after parental RNAi”.

Significance (Line 70-73): “RNA interference of iPTHR resulted in defects in wing exoskeleton maturation and fecundity. Based on the differential gene expression patterns and the phenotype induced by RNAi, we propose that the iPTH system is likely involved in the regulation of exoskeletal cuticle formation and fecundity in insects.”

Fig. 6 (Line 279-280): RNAi resulting in suppression of the transcript levels with wing and fecundity phenotypes.

9. Acronyms are not adequately defined in many instances; e.g. CPR family (line 325), DE analyses (line 512).

**Response:** Line 336-337: The full name of CPR, “Cuticular Protein with the Rebers and Riddiford Consensus” has been added in the text.

Line 406-407: We also added the full name of CPAP, “Cuticular Proteins Analogous to Peritrophins” in the text.

“DE” in “DE analyses” is the abbreviation of “differentially expressed”, here we defined it in Line 303 when it first appeared in the text, and then used “DE” in the following parts in the text.

10. Abstract, line 39-40. Reference formatting is inconsistent and it is unusual to cite references in the abstract.

**Response:** Line 40: We have removed the reference in the abstract.

11. Lines 158-159. Fig 2B contains no information to support this statement of negative results with untransfected cells.

**Response:** Line 174-175: Negative result was explained in the text: “The cells without the Tc-iPTHR constructs did not respond to the Tc-iPTH at 10 µM”. The lack of response is not shown on the figure, but the text explained it.

12. The manuscript requires further editing to correct English grammar and spelling.

**Response:** The revision was carefully reviewed for English grammar and spelling.

References

1.	Harmar AJ. Family-B G-protein-coupled receptors. Genome Biol. 2001;2(12):REVIEWS3013.

2.	Tanaka Y, Suetsugu Y, Yamamoto K, Noda H, Shinoda T. Transcriptome analysis of neuropeptides and G-protein coupled receptors (GPCRs) for neuropeptides in the brown planthopper Nilaparvata lugens. Peptides. 2014;53:125-33.

3.	Wilmen A, Goke B, Goke R. The isolated N-terminal extracellular domain of the glucagon-like peptide-1 (GLP)-1 receptor has intrinsic binding activity. FEBS Lett. 1996;398(1):43-7.

4.	Gaudin P, Couvineau A, Maoret JJ, Rouyer-Fessard C, Laburthe M. Mutational analysis of cysteine residues within the extracellular domains of the human vasoactive intestinal peptide (VIP) 1 receptor identifies seven mutants that are defective in VIP binding. Biochem Biophys Res Commun. 1995;211(3):901-8.

5.	Qi LJ, Leung AT, Xiong Y, Marx KA, Abou-Samra AB. Extracellular cysteines of the corticotropin-releasing factor receptor are critical for ligand interaction. Biochemistry. 1997;36(41):12442-8.

6.	Karageorgos V, Venihaki M, Sakellaris S, Pardalos M, Kontakis G, Matsoukas MT, et al. Current understanding of the structure and function of family B GPCRs to design novel drugs. Hormones (Athens). 2018;17(1):45-59.

7.	Kulkarni MM, Booker M, Silver SJ, Friedman A, Hong P, Perrimon N, et al. Evidence of off-target effects associated with long dsRNAs in Drosophila melanogaster cell-based assays. Nat Methods. 2006;3(10):833-8.

8.	Kalsi M, Palli SR. Cap n collar transcription factor regulates multiple genes coding for proteins involved in insecticide detoxification in the red flour beetle, Tribolium castaneum. Insect Biochem Mol Biol. 2017;90:43-52.

9.	Tabunoki H, Gorman MJ, Dittmer NT, Kanost MR. Superoxide dismutase 2 knockdown leads to defects in locomotor activity, sensitivity to paraquat, and increased cuticle pigmentation in Tribolium castaneum. Sci Rep. 2016;6:29583.

10.	Kim HG, Margolies DC, Park Y. Roles of transient receptor potential channels in eclosion and movement in the res flour beetle Tribolium castaneum. Physiological Entomology. 2018;43:79-85.

11.	Aikins MJ, Schooley DA, Begum K, Detheux M, Beeman RW, Park Y. Vasopressin-like peptide and its receptor function in an indirect diuretic signaling pathway in the red flour beetle. Insect Biochem Mol Biol. 2008;38(7):740-8.

12.	Beets I, Janssen T, Meelkop E, Temmerman L, Suetens N, Rademakers S, et al. Vasopressin/oxytocin-related signaling regulates gustatory associative learning in C. elegans. Science. 2012;338(6106):543-5.

13.	Van Sinay E, Mirabeau O, Depuydt G, Van Hiel MB, Peymen K, Watteyne J, et al. Evolutionarily conserved TRH neuropeptide pathway regulates growth in Caenorhabditis elegans. Proc Natl Acad Sci U S A. 2017;114(20):E4065-E74.

14.	Li B, Predel R, Neupert S, Hauser F, Tanaka Y, Cazzamali G, et al. Genomics, transcriptomics, and peptidomics of neuropeptides and protein hormones in the red flour beetle Tribolium castaneum. Genome Res. 2008;18(1):113-22.

15.	Suarez-Bregua P, Cal L, Canestro C, Rotllant J. PTH Reloaded: A New Evolutionary Perspective. Front Physiol. 2017;8:776.

16.	Mirabeau O, Joly JS. Molecular evolution of peptidergic signaling systems in bilaterians. Proc Natl Acad Sci U S A. 2013;110(22):E2028-37.

17.	Jekely G. Global view of the evolution and diversity of metazoan neuropeptide signaling. Proc Natl Acad Sci U S A. 2013;110(21):8702-7.

18.	Elphick MR, Mirabeau O, Larhammar D. Evolution of neuropeptide signalling systems. J Exp Biol. 2018;221(Pt 3).

19.	Copenhaver PF, Taghert PH. Neurogenesis in the insect enteric nervous system: Generation of pre-migratory neurons from an epithelial placode Development. 1990;109:17-28.

20.	Zhu Q, Arakane Y, Beeman RW, Kramer KJ, Muthukrishnan S. Functional specialization among insect chitinase family genes revealed by RNA interference. Proc Natl Acad Sci U S A. 2008;105(18):6650-5.

21.	Zhu Q, Arakane Y, Beeman RW, Kramer KJ, Muthukrishnan S. Characterization of recombinant chitinase-like proteins of Drosophila melanogaster and Tribolium castaneum. Insect Biochem Mol Biol. 2008;38(4):467-77.

22.	Zhu Q, Arakane Y, Banerjee D, Beeman RW, Kramer KJ, Muthukrishnan S. Domain organization and phylogenetic analysis of the chitinase-like family of proteins in three species of insects. Insect Biochem Mol Biol. 2008;38(4):452-66.

23.	On JS, Chow BK, Lee LT. Evolution of parathyroid hormone receptor family and their ligands in vertebrate. Front Endocrinol (Lausanne). 2015;6:28.

24.	Pinheiro PL, Cardoso JC, Power DM, Canario AV. Functional characterization and evolution of PTH/PTHrP receptors: insights from the chicken. BMC Evol Biol. 2012;12:110.
