# Supplementary material for: A new neuropeptide insect parathyroid hormone iPTH in the red flour beetle Tribolium castaneum
Source: PLoS Genet. 2020 May 4;16(5):e1008772. doi: 10.1371/journal.pgen.1008772 (PMC7224569; doi:10.1371/journal.pgen.1008772)
Supplement: Attachment — Submitted filename: Response to reviewers.docx [file pgen.1008772.s016.docx]

Comments to the Authors:

Please note here if the review is uploaded as an attachment.

Reviewer #1: Several comments have been well addressed, but some are remaining:

**Response:** We thank the reviewer for the constructive criticisms and we have revised the manuscript according to your new suggestions.

Introduction

Line[97-98]: Described references are not added in the text, nor added in reference list. Please do so, check more carefully …

**Response:** The two described references are added in the text and reference list in the manuscript as,

Text: Line 93-96 (Manuscript file)/ Line 93-96 (Related manuscript file).

“our previous study has described two receptors in the red flour beetle, *Tribolium castaneum*, as an orthologous group similar to the PTH-receptors of vertebrates in the G protein-coupled receptor (GPCR) B group [13, 14]. The ligand for these receptors has not yet been identified [15].”

Reference list: Line 615-618 (Manuscript file)/ Line 622-625 (Related manuscript file).

“14.	Harmar AJ. Family-B G-protein-coupled receptors. Genome Biol. 2001;2(12):REVIEWS3013.

15.	Tanaka Y, Suetsugu Y, Yamamoto K, Noda H, Shinoda T. Transcriptome analysis of neuropeptides and G-protein coupled receptors (GPCRs) for neuropeptides in the brown planthopper Nilaparvata lugens. Peptides. 2014; 53:125-33.”

Line[161] Add “,” between … binding pocket - and - as stabilization…

**Response:** Line 163 (Manuscript file), we add a “,” between … binding pocket - and - as stabilization…

Discussion:

[441-445]: sentences are incoherent, revise wording.

**Response:** Line 441-448 (Manuscript file) is now revised as below,

“In summary, we identified a new insect peptidergic system, iPTH, which is the orthologous system to vertebrate PTH, showing that the PTH system is an ancestral signaling system dating back to the evolutionary time before the divergence of protostomes and deuterostomes, although it has been lost in Diptera and Lepidoptera. High expression at both mRNA and protein levels of the iPTH and its receptors in the gut and the CNS of *Tribolium* indicated that this neuropeptide may have important regulatory functions. We investigated this by RNAi and showed that iPTH system plays an essential role in cuticle maturation and fecundity in insects.”

More in depth: L442: Too vague: high expression of what? RNA, protein? Be more specific.

**Response:** It showed high expression at both mRNA and protein level in the gut and the CNS. Thus, we added “at both mRNA and protein levels” to the sentence to make it more specific in Line 445 (Manuscript file).

L443: Too vague: which additional functions? This does not add anything. Either describe which additional functions you imply, or don’t mention this.

**Response:** Line 445-448 (Manuscript file), we removed the description of “additional functions” and changed the sentences as below: “High expression at both mRNA and protein levels of the iPTH and its receptors in the gut and the CNS of *Tribolium* indicated that this neuropeptide may have important regulatory functions. We investigated this by RNAi and showed that iPTH system plays an essential role in cuticle maturation and fecundity in insects.”

[354-355]: Figure shows exactly what was asked for, but it should be shown to readers in order to make these claims. Add this figure either to Fig 7 or Fig 8 as an extra panel. Or if not shown, than the claim that the receptors are unable to compensate each other should be eliminated from your text.

**Response:** We added this figure as Fig. 6D, and rearranged Fig. 6.

Line 259-261 (Manuscript file), according to the rearranged Fig. 6, we changed the sentence to “Both larval RNAi and pupal RNAi of *Tc-iPTHR1* and *Tc-iPTHR2*, which successfully suppressed the expression of their specific target genes (Fig. 6A and D), resulted in partial defects in adult eclosion” (Fig. 6B and C).

Reviewer #2: I thank the authors for taking into account my remarks which I consider have been sufficiently addressed.

Some of the newly written sentences could be improved:

**Response:** We appreciate the reviewer’s useful comments to improve our manuscript, and we revised the manuscript again following your new suggestions as bellow：

lines 58-59: Vertebrate parathyroid hormone (PTH) and its receptors have been extensively studied with RESPECT TO [the] THEIR function [of] IN bone remodeling and calcium metabolism.

**Response:** Line 58-59 (Manuscript file), the sentence has been revised to: “Vertebrate parathyroid hormone (PTH) and its receptors have been extensively studied with respect to their function in bone remodeling and calcium metabolism.”

lines 53-54: Moreover, RNAi of iPTHRs also led TO significant reductions in egg numbers and hatching rates after parental RNAi.

**Response:** Line 52-53 (Manuscript file), “to” was added after “led”.

line 59-61: Insect parathyroid hormone receptors (iPTHRs) [were] HAVE BEEN previously described as counterparts of vertebrate PTHRs.

**Response:** Line 60-61 (Manuscript file), “were” has been changed to “have been”.

line 82-83: Functional studies of ancestral bilaterian neuropeptides have been successful by starting from the sequence similarities, e.g., vasopressin AND TRH [1-4]

**Response:** Line 81-83 (Manuscript file), the sentence has been revised to: “Functional studies of ancestral bilaterian neuropeptides have been successful by starting from the sequence similarities, e.g., vasopressin and thyrotropin-releasing hormone”.

line150: coincided with the [lack] ABSENCE of this specific

**Response:** Line 104-105 (Manuscript file), it has been changed to “coincided with the absence of this specific neuropeptide in the same taxa”.

Line 146-147 (Manuscript file), it has been changed to “consistent with the absence of iPTH sequence in those taxa”.

line 152: Maximum likelihood method with THE JTT model

**Response:** Line 150, Line 466-467, and Line 741 (Manuscript file), “the” has been added before “JTT model”.

line 220: neuronal cells and axonal projections in THE CNS

**Response:** Line 217 (Manuscript file), “the” has been added before “CNS”.

line 284: beetles arrested at the time of [ecolsion] ECLOSION

**Response:** Line 281 (Manuscript file), the word has been corrected to “eclosion”.

line 297: suppression of Tc-iPTH by the RNAi may [have not] NOT HAVE been sufficient to induce

**Response:** Line 293-294 (Manuscript file), the sentence has been revised to “suppression of Tc-iPTH by the RNAi may not have been sufficient to induce…”

lines 306-307: plots also showed THAT DE genes in the dsiPTHR1 and dsiPTHR2 treatments [to] have similar patterns

**Response:** Line 302-303 (Manuscript file), the sentence has been revised to “plots also showed that DE genes in the ds*iPTHR1* and ds*iPTHR2* treatments have similar patterns”.

line 310: the correlation between dsiPTHR1 and dsiPTHR2 treatments [with a R-square showing 0.78] was obvious (R-SQUARED=0.78)

**Response:** Line 304-305 (Manuscript file), the sentence has been revised to “the correlation between ds*iPTHR1* and ds*iPTHR2* treatments was obvious (R^2^ = 0.78)”.

line 430: The CONCLUSIONS DRAWN FROM THE DE data ANALYSIS [together] were also supported by cuticle contents

**Response:** Line 408-409 (Manuscript file), the sentence has been revised to “The conclusions drawn from the DE data analysis were also supported by cuticle contents…”

line 460: [yet] STILL displayed [remaining] RESIDUAL iPTH immunoreactivity

**Response:** Line 436 (Manuscript file), “yet” has been changed to “still”, and “remaining” has been changed to “residual”.

line 472: THAT THIS NEUROPEPTIDE MAY HAVE additional functions.

**Response:** Line 445-447 (Manuscript file), combined with the comment of another reviewer, the sentence has been revised to “High expression at both mRNA and protein levels of the iPTH and its receptors in the gut and the CNS of *Tribolium* indicated that this neuropeptide may have important regulatory functions.”

line 805: "Two-dimensional scatter plots" instead of "Two dimensional scatter plots"

**Response:** Line 301-302, Line 313-314 and Line 769 (Manuscript file), “-” has been added between "Two” and “dimensional”, or between “Three” and “dimensional”.
